# Differential expression of *HAVCR2* gene in pan-cancer: A potential biomarker for survival and immunotherapy

**DOI:** 10.3389/fgene.2022.972664

**Published:** 2022-08-23

**Authors:** Hetong Li, Dinglong Yang, Min Hao, Hongqi Liu

**Affiliations:** ^1^ Second Clinical Medical College, Shanxi Medical University, Taiyuan, China; ^2^ Department of Obstetrics and Gynecology, The Second Hospital of Shanxi Medical University, Taiyuan, China; ^3^ Department of Information Management, The Second Hospital of Shanxi Medical University, Taiyuan, China

**Keywords:** *HAVCR2*, TIM-3, immune checkpoints, immunotherapy, pan-cancer

## Abstract

T-cell immunoglobulin mucin 3 (TIM-3) has emerged as a promising immune checkpoint target in cancer therapy. However, the profile of the hepatitis A virus cellular receptor 2 (*HAVCR2*) gene, encoding TIM-3 expression, is still obscure, along with its role in cancer immunity and prognosis. This study comprehensively analyzed *HAVCR2* expression patterns in pan-cancer and underlined its potential value for immune checkpoint inhibitor-based immunotherapy. Our results displayed that *HAVCR2* was differentially expressed and closely corresponded to survival status in pan-cancer. More importantly, the *HAVCR2* expression level was also significantly related to cancer immune infiltration, immune checkpoint genes, and immune marker genes. Enrichment analyses implicated *HAVCR2*-associated terms in cancer, including immunity, metabolism, and inflammation. Our study demonstrated that *HAVCR2* could participate in differing degrees of immune infiltration in tumorigenesis. The highlights of the *HAVCR2* pathway revealed that TIM-3 could function as both a biomarker and clinical target to improve the therapeutic efficacy of immunotherapy.

## Introduction

Cancer is a leading cause of death, accounting for 13% of all humans (Bray et al., 2018; Sung et al., 2021). Cancer burden, morbidity, and mortality are increasing at a high rate of speed, having an alarming impact globally. Strategies in the clinical setting for cancer treatment include chemotherapy, irradiation, surgery, and immunotherapy. Immunotherapy is a revolutionized treatment for cancers. The cytotoxic T-lymphocyte-associated antigen-4 (CTLA-4) and programmed cell death protein 1 (PD-1) are two common targeted immune checkpoint inhibition pathways that have achieved durable responses (Qin et al., 2019). However, the success rate of immune checkpoint inhibition is not ideal, and there are still abundant cancers refractory to CTLA-4 and PD-1 blockade, like colon adenocarcinoma (COAD) (Das et al., 2017; O'Connell et al., 2021). This has provided impetus to identify new checkpoint targets or combine other co-inhibitory receptors that could further improve response rates of current immunotherapeutic drugs and achieve responses to cancer types resistant to immunotherapy.

*HAVCR2* (hepatitis A virus cellular receptor 2) located on 5q33.2 encodes T-cell immunoglobulin mucin 3 (TIM-3) protein, which is a potential immune-checkpoint target in tumors (Wolf et al., 2020). The role of *HAVCR2* has been found in subcutaneous panniculitis-like T-cell lymphoma (SPTCL), COAD, and esophageal carcinoma (ESCA) (O'Connell et al., 2021; Cui et al., 2021; Sonigo et al., 2020). Research studies have investigated *HAVCR2* mRNA and protein expressions are significantly upregulated across diverse cancers and associated with poor prognosis in cervical squamous cell carcinoma and endocervical adenocarcinoma (CESC), kidney renal clear cell carcinoma (KIRC), COAD, bladder urothelial carcinoma (BLCA), and stomach adenocarcinoma (STAD) (Cao et al., 2013; Jiang et al., 2013; Yuan et al., 2014; Yang et al., 2015; Zhou et al., 2015). Previous studies about TIM-3 concentrated on regulation in autoimmunity such as immune thrombocytopenia, multiple sclerosis, and systemic lupus erythematosus (Joller et al., 2012; Zhang and Shan, 2014). TIM-3 has recently gained attention in cancer due to its persistent antigen T-cell stimulation (Sakuishi et al., 2010; Ngiow et al., 2011). Checkpoint receptors on T-cell surface can limit T-cell activation to regulate immune responses and show the potential as drug targets. TIM-3 is a checkpoint receptor expressed on activated Th1 cell surface acting as a negative regulator to T-cell death and plays a role by interacting with its ligand galectin-9 (Gal-9) (Li et al., 2021). Apart from acting as mediators of T helper (Th) 1 cells, TIM-3 could inhibit both innate and adaptive immune responses related to the cluster of differentiation CD8^+^ T and Th17 cells and tolerance induction associated with regulatory T cells (Tregs), resulting in suppressing immune response (Monney et al., 2002; Tang et al., 2019).

Nevertheless, most reports about *HAVCR2* were limited to a specific cancer type. Pan-cancer analysis can identify common features and heterogeneities in cancer processes. The present study comprehensively analyzed the expression, prognosis, functions, and pathways of *HAVCR2* in pan-cancer and unveiled its potential application in immune treatment.

Our study analyzed *HAVCR2* expression patterns and prognosis in multiple types of malignancy. We further assessed diagnosis value, genetic alteration, epigenetic characteristics, correlation with tumor immunity, and signal pathways that are associated with the *HAVCR2* gene. The results of this study were conducive to understanding the functional role of *HAVCR2* in the context of tumors and illustrated the potential mechanism of *HAVCR2* with tumor–immune interactions, highlighting a potential candidate and biomarker for the immunotherapy revolution in pan-cancer.

## Materials and methods

### Date acquisition

The differential expression of *HAVCR2* between pan-cancer and matched standard samples was extracted with the combination of the sample data from the Genotype-Tissue Expression (GTEx) and The Cancer Genome Atlas (TCGA) databases. Clinical characteristics including gender, age, and tumor node metastasis (TNM) stages were downloaded from the TCGA database. Then, RNA-seq was transformed into TPM (transcripts per million reads) for the following analysis. All expression data were Log2 transformed.

### Diagnostic and prognostic analyses

The diagnostic value of *HAVCR2* was assessed using the area under the curve (AUC) performance of the receiver operating characteristic (ROC) analysis. The AUC greater than 0.8 is considered to be of great diagnostic value. The ggplot2 R package (version 3.3.3) and pROC R package (version 1.17.0.1) were used to make ROC curves. Survival curves were drawn using the R packages “survival” (version 3.2.10) and “survminer” (version 0.4.9) to analyze the survival differences between low and high expression groups in each type of cancer patients according to *HAVCR2* expression. For prognostic value, common endpoints were employed including disease-specific survival (DSS), progression-free interval (PFI), and overall survival (OS) using forest plots. The hazard ratios with 95% confidence intervals were calculated using univariate survival analysis. Kaplan–Meier curves and a log-rank test were used to analyze the relationship between the survival time and *HAVCR2* expression stratified at high or low levels.

### Relationship between *HAVCR2* expression and immunity

The RNA-Seq expression profile and corresponding tissues of 39 types of cancer data were obtained to analyze the correlation between *HAVCR2* expression and five types of immune cell scores (B cells, T cells, neutrophils, dendritic cells, and macrophages). For further immune infiltration evaluation, we used GSVA, which is an R software package (version 1.34.0) that could compute the connection between *HAVCR2* expression and immune cell enrichment in pan-cancer (Hanzelmann et al., 2013). The ESTIMATE method was used to analyze immune infiltration in tumor tissues, including stromal and immune cells based on gene expression profiles (Li et al., 2020). The correlation coefficient between the *HAVCR2* gene and immune checkpoint genes was tested with Spearman’s rank correlation.

### *HAVCR2* gene expression and immune markers

The co-expression analysis was conducted in high and low *HAVCR2* expressions with microsatellite instability (MSI), tumor mutational burden (TMB), immune neoantigens, DNA methylation, and DNA repair genes in the tumor group. DNA methylation analysis was based on Illumina methylation 450 data and the cg09574807 probe. Spearman’s rank correlation test was applied to obtain *p*-values and correlation values.

### Relationship between *HAVCR2* expression and clinical characteristics

After dividing patients into different groups according to gender (male and female), age (median), and pathological stages (stage I, stage II, stage III, and stage IV), RNA-sequencing expression profiles for *HAVCR2* in different types of cancer were obtained from the TCGA database. The log2 [TPM +1]-transformed expression data were applied for the box plots. The box plots showed data including minimum, maximum, first quartile, third quartile, and median. All analyses were implemented by R (version 4.0.3).

### KEGG and GSEA

The Kyoto Encyclopedia of Genes and Genomes (KEGG) database and Gene Set Enrichment Analysis (GSEA) were used to analyze the enrichment of *HAVCR2* gene expression in signaling pathways. R package clusterProfiler (3.14.3) was used to achieve enrichment maps (Yu et al., 2012). The adjusted *p*-value (<0.05), normalized enrichment score (|NES| > 1), and FDR q-value (<0.25) were used to classify enrichment differences of function in each phenotype.

### Immunohistochemical staining

IHC images of *HAVCR2* protein expression in normal and tumor tissues were explored in The Human Protein Atlas (HPA: https://www.proteinatlas.org/) database. Sangerbox (http://sangerbox.com/) tool was used to visualize subcellular locations of *HAVCR2*.

### Statistical analysis

*HAVCR2* expression comparison was estimated using the Wilcoxon rank sum test and Kruskal–Wallis test. The prognostic value of *HAVCR2* gene in 39 tumors was forecasted by dichotomizing *HAVCR2* expression into high and low expression levels (Liu et al., 2018). Survival analysis utilized univariate Cox regression analysis to calculate the hazard ratio (HR) and *p*-value. R language (version 4.0.3) was used for all statistical analyses. The threshold of *p* < 0.05 indicated a statistically significant difference (∗p < 0.05, ∗∗p < 0.01, and ∗∗∗*p* < 0.001).

## Results

### Pan-cancer expression landscape of *HAVCR2*

**FIGURE 1:**
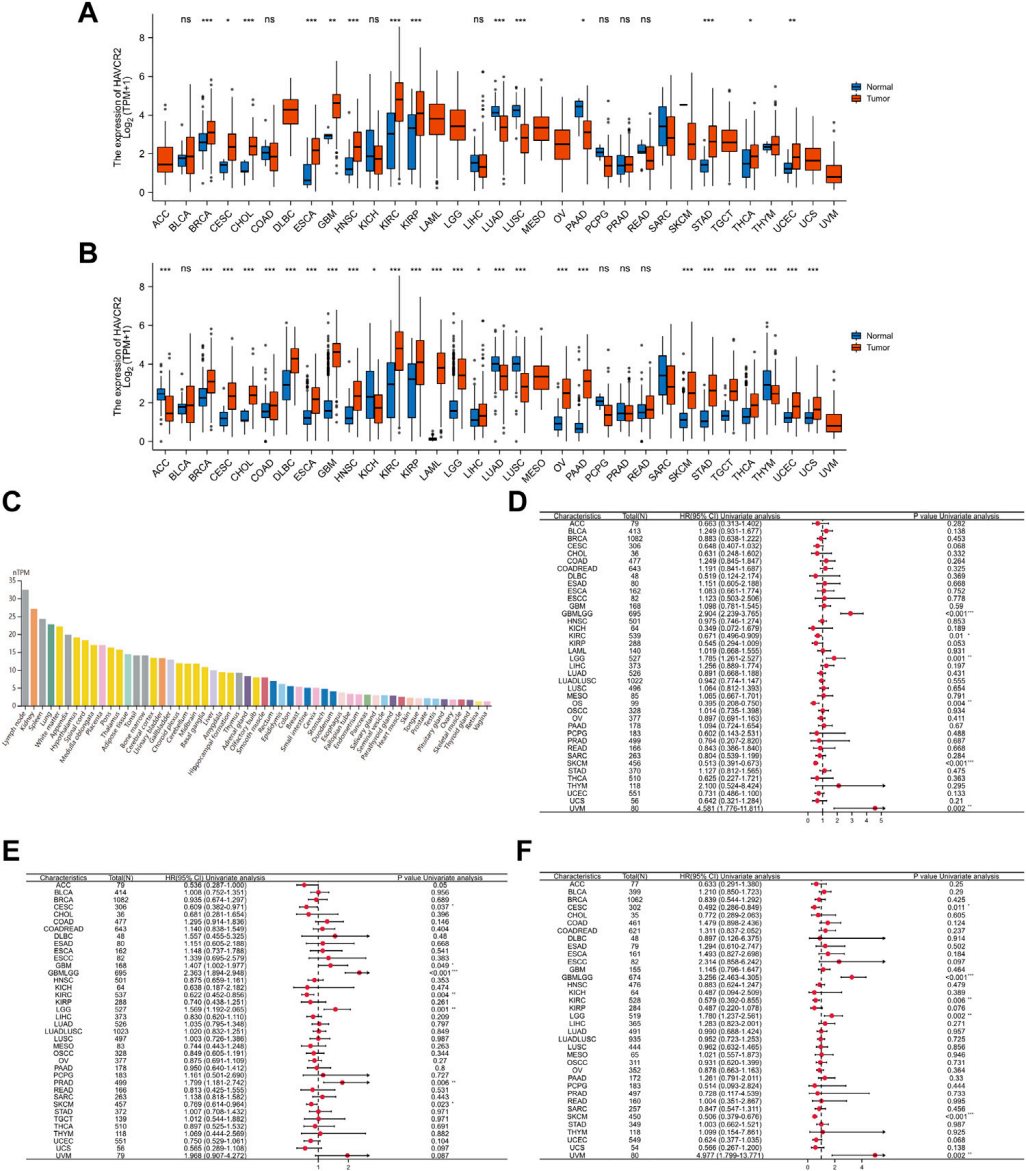
Comprehensive analysis of *HAVCR2* expression in pan-cancer. **(A)** Human *HAVCR2* expression levels in different cancer types from TCGA data. **(B)** Differential expression of *HAVCR2* in different cancer tissues compared with normal tissues in TCGA-combined GTEx data. **(C)** RNA level of *HAVCR2* is the highest in the lymph node, while lowest in the vagina for human normal tissues in the Consensus dataset. **(D–F)** Forest maps showed the relationship between *HAVCR2* expression and OS **(D)**, PFI **(E)**, and DSS **(F)** in pan-cancer. The symbols “*,” “**,” and “***” refer to *p*-values <0.05, <0.01, and <0.001, respectively.

Tumor samples from TCGA were first assessed, and we found relatively higher *HAVCR2* gene expression in breast invasive carcinoma (BRCA), CESC, cholangiocarcinoma (CHOL), ESCA, glioblastoma multiforme (GBM), head and neck squamous cell carcinoma (HNSC), KIRC, kidney renal papillary cell carcinoma (KIRP), thyroid cancer (THCA), stomach adenocarcinoma (STAD), and uterine corpus endometrial carcinoma (UCEC). In comparison, low *HAVCR2* expression was observed in lung adenocarcinoma (LUAD), pancreatic adenocarcinoma (PAAD), and lung squamous cell carcinoma (LUSC) (Figure 1A). Furthermore, the data from TCGA and GTEx databases were combined for expression analysis to ensure a more reliable result (Figure 1B). *HAVCR2* expression was remarkably increased in 21 tumor types: BRCA, CESC, COAD, ESCA, CHOL, HNSC, lymphoid neoplasm diffuse large B-cell lymphoma (DLBCL), GBM, KIRC, KIRP, acute myeloid leukemia (AML), brain lower-grade glioma (LGG), liver hepatocellular carcinoma (LIHC), PAAD, ovarian serous cystadenocarcinoma (OV), THCA, testicular germ cell tumors (TGCT), UCEC, skin cutaneous melanoma (SKCM), STAD, and uterine carcinoma (UCS). However, low *HAVCR2* expression was observed in five tumor types: adrenocortical carcinoma (ACC), kidney chromophobe (KICH), LUAD, LUSC, and thymoma (THYM). In addition, we continued to evaluate *HAVCR2* RNA expression in human normal tissues and found it was highest in the lymph node, while lowest in the vagina in the Consensus dataset (Figure 1C).

### Prognostic value of *HAVCR2* gene in pan-cancer

Three indicators, OS, PFI, and DSS, were used to judge the prognostic value. Results exhibiting bad or good prognosis are reflected on mRNA abundance to clarify the *HAVCR2* effect. Cox regression analysis of OS identified that *HAVCR2* was markedly correlated with the prognosis of glioma (GBMLGG) (*p <* 0.001), KIRC (*p =* 0.01), LGG (*p =* 0.001), osteosarcoma (*p =* 0.004), SKCM (*p <* 0.001), and uveal melanoma (UVM) (*p =* 0.002) (Figure 1D). PFI displayed that *HAVCR2* was notably related to the prognosis of CESC (*p =* 0.037), GBM (*p =* 0.049), GBMLGG (*p <* 0.001), KIRC (*p =* 0.004), LGG (*p =* 0.001), PRAD (*p =* 0.006), and SKCM (*p =* 0.023) (Figure 1E). DSS reflected that *HAVCR2* was correlated with the prognosis of CESC (*p =* 0.011), GBMLGG (*p <* 0.001), KIRC (*p =* 0.006), LGG (*p =* 0.002), SKCM (*p <* 0.001), and UVM (*p =* 0.002) (Figure 1F).

**FIGURE 2:**
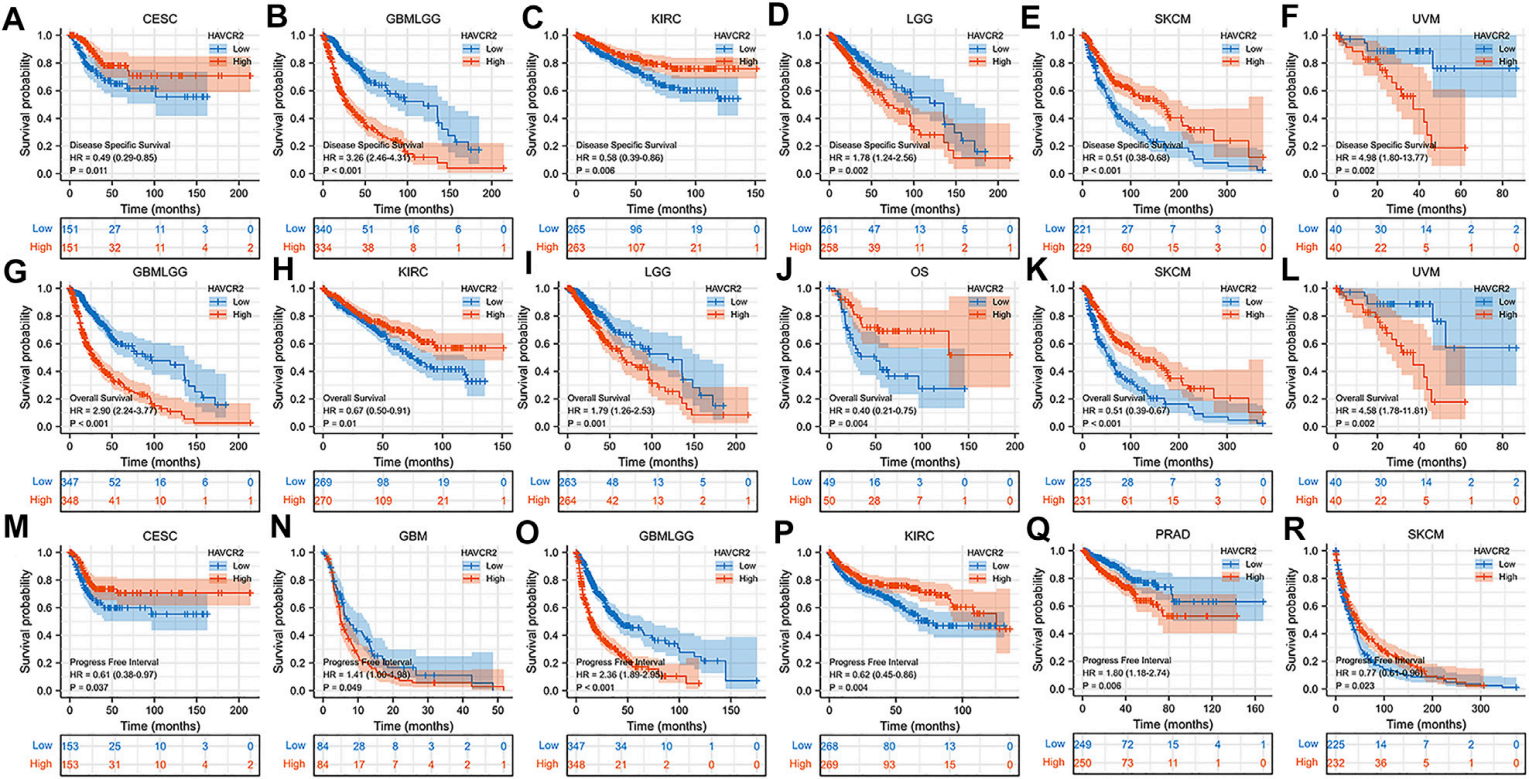
Kaplan–Meier survival curves with a significant correlation between high and low *HAVCR2* gene expressions of cancer types in DSS **(A–F)**, OS **(G–L),** and PFI **(M–R)**.

We further determined the effect of aberrant *HAVCR2* expression on prognosis by using Kaplan–Meier plotter in pan-cancer. Our results displayed that *HAVCR2* had multifaceted prognostic values of disease-specific survival, overall survival, and progress-free survival in different types of cancer (Figure 2). These outcomes indicated that *HAVCR2* expression was a protective factor in CESC (Figures 2A,M), KIRC (Figures 2C,H,P), SKCM (Figures 2E,K,R), and osteosarcoma (Figure 2J). In contrast, *HAVCR2* predicted worse prognosis in GBM (Figure 2N), GBMLGG (Figures 2B,G,O), LGG (Figures 2D,I), PRAD (Figure 2Q), and UVM (Figures 2F,L).

### Diagnostic value of *HAVCR2* in cancer

**FIGURE 3:**
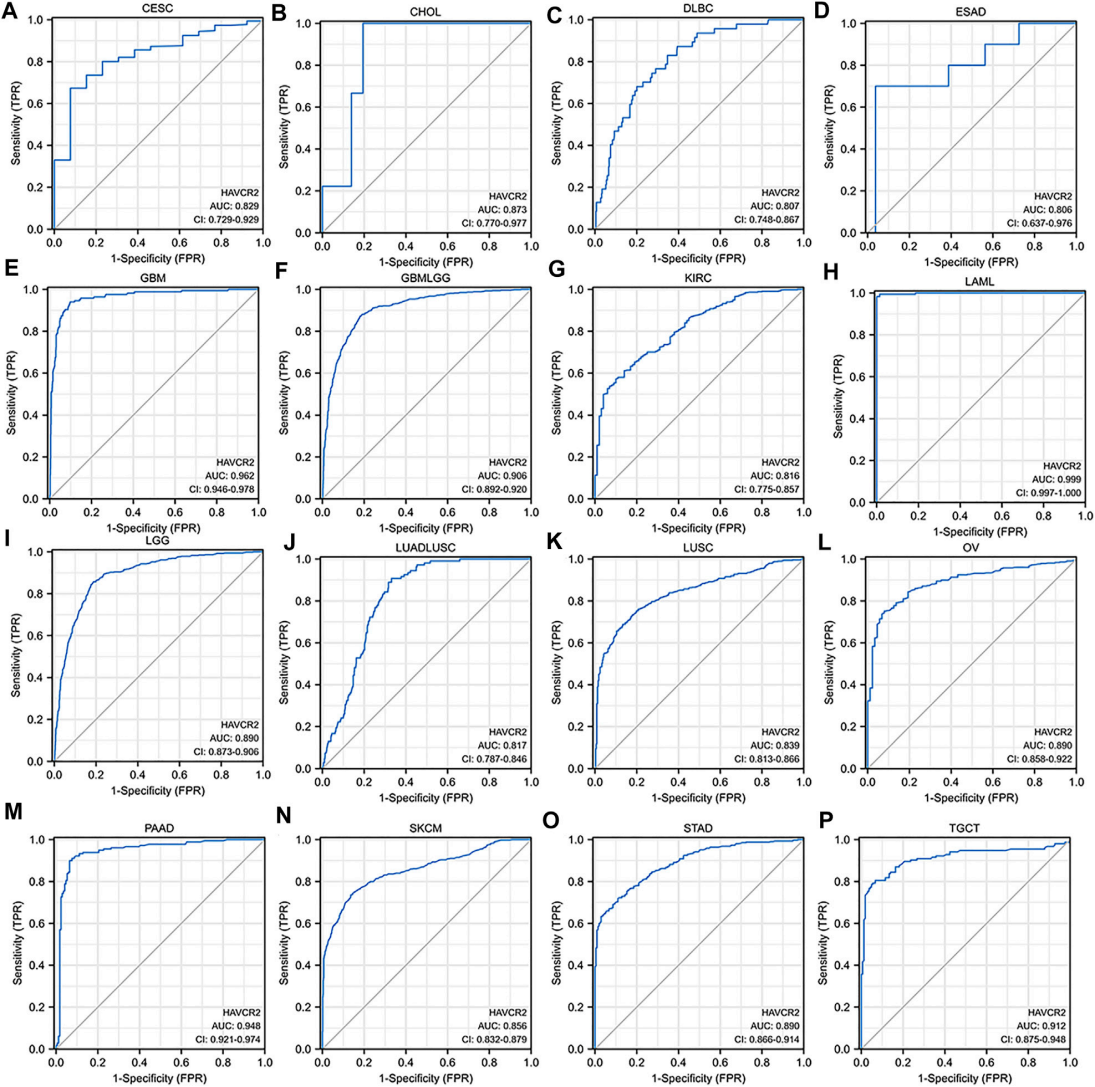
Receiver operating characteristic (ROC) curves for *HAVCR2* in pan-cancer **(A–P)**. The ROC curves with the area under the curve (AUC) value more than 0.8 were considered significant for cancer diagnosis. *HAVCR2* gene has a high diagnosis value with AUC greater than 0.9 in **(E)** GBM (AUC = 0.962), **(F)** GBMLGG (AUC = 0.906), **(H)** LAML (AUC = 0.999), **(M)** PAAD (AUC = 0.948), and **(P)** TGCT (AUC = 0.912).

We assessed the diagnostic value of *HAVCR2* using ROC curves in pan-cancer. The ROC curves with AUC greater than 0.8 are included in Figure 3. The AUC of five cancer types including LAML (AUC = 0.999) (Figure 3H), GBM (AUC = 0.962) (Figure 3E), PAAD (AUC = 0.948) (Figure 3M), TGCT (AUC = 0.912) (Figure 3P), and GBMLGG (AUC = 0.906) (Figure 3F) was greater than 0.9. In addition, 10 cancer types including CESC (AUC = 0.829) (Figure 3A), CHOL (AUC = 0.873) (Figure 3B), DLBC (AUC = 0.807) (Figure 3C), ESAD (AUC = 0.806) (Figure 3D), KIRC (AUC = 0.816) (Figure 3G), LGG (AUC = 0.890) (Figure 3I), LUSC (AUC = 0.839) (Figure 3K), OV (AUC = 0.890) (Figure 3L), SKCM (AUC = 0.856) (Figure 3N), and STAD (AUC = 0.890) (Figure 3O) were with AUC greater than 0.8. For LUAD, *HAVCR2* did not show prognostic values, but when combined with LUSC, multivariate analysis showed a significant AUC of 0.817 (Figure 3J).

### Clinicopathological characteristics of *HAVCR2* expression

**FIGURE 4:**
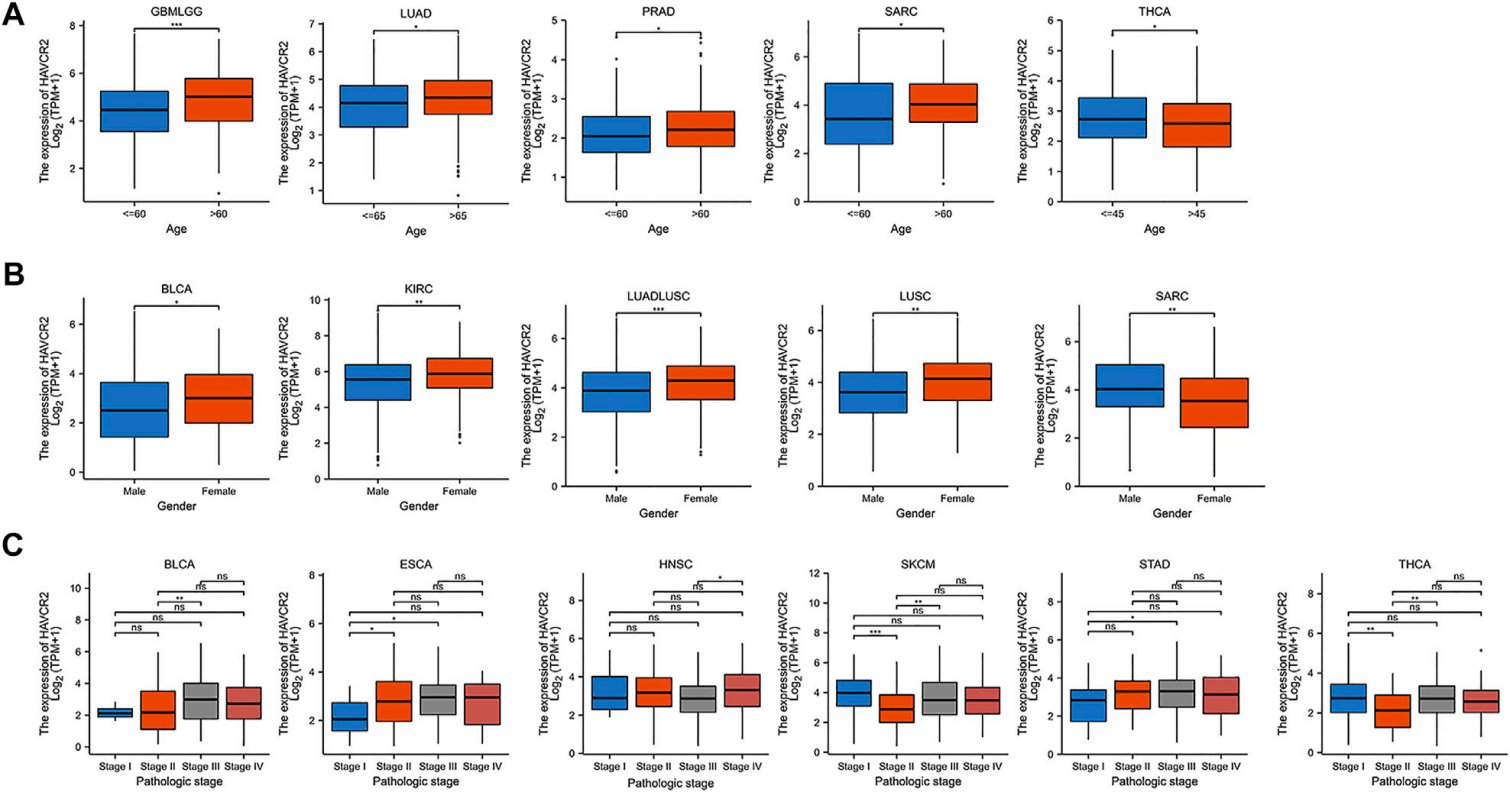
Pan-cancer *HAVCR2* expression in different age, gender, and TNM stages. **(A)** *HAVCR2* expression was correlated with age in GBMLGG, LUAD, PRAD, SARC, and THCA. **(B)** *HAVCR2* expression was related to gender in BLCA, KIRC, LUADLUSC, LUSC, and SARC. **(C)** *HAVCR2* expression was associated with BLCA, ESCA, HNSC, STAD, SKCM, and THCA. *p < 0.05, **p < 0.01, and ***p < 0.001.

We further investigated the association of *HAVCR2* expression and age, gender, and tumor node metastasis (TNM) stages. Baseline characteristics of pan-cancer patients were obtained from TCGA (Supplementary Table S1). We found that it was higher in older ages in most tumors including GBMLGG, LUAD, PRAD, and SARC, while *HAVCR2* expression was lower in THCA in older ages (Figure 4A). Higher *HAVCR2* expression of females was observed in BLCA, KIRC, LUADLUSC, and LUSC than in males. In contrast, higher *HAVCR2* expression was in male in SARC (Figure 4B). We also investigated different tumor node metastasis stages and found that it was higher in late stages in BLCA, ESCA, HNSC, and STAD and in early stages of SKCM and THCA (Figure 4C).

### *HAVCR2* level related to immune infiltration

**FIGURE 5:**
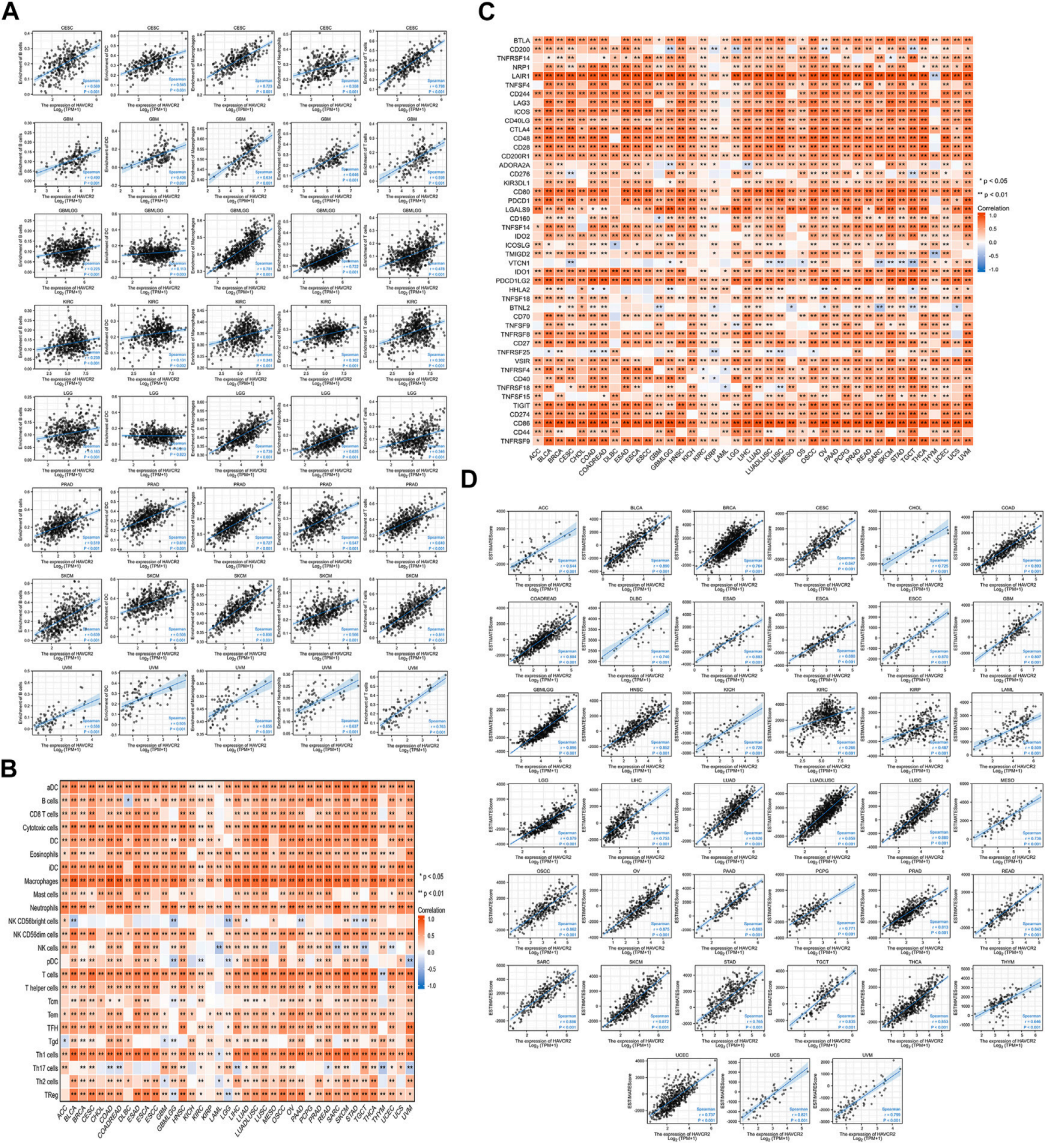
Correlation between *HAVCR2* gene expression and immune infiltration, ESTIMATE score, and immune checkpoints in pan-cancer. **(A)** *HAVCR2* expression level had significant positive correlations with the infiltration levels of five types of infiltrating immune cells in eight prognosis-related cancers. **(B)** Heatmap representing *HAVCR2* expression was found to be substantially linked with the infiltration levels of 24 immune infiltrating cells in pan-cancer. **(C)** Correlation between *HAVCR2* expression and immune checkpoint genes in pan-cancer. **(D)** *HAVCR2* expression was positively and significantly correlated with ESTIMATE score in all 39 cancers (r > 0 and ***p < 0.001).

Eight tumors with a significant prognosis value of *HAVCR2* were further analyzed for exploring the relationship with immune cell infiltration. B cells, dendritic cells, T cells, macrophages, and neutrophils participated in immune responses in cancer immunity extensively (Lei et al., 2020). The results suggested *HAVCR2* expression had significant positive correlations with the infiltration of the aforementioned five types of infiltrating immune cells in seven prognosis-related cancers, including CESC, GBM, GBMLGG, KIRC, PRAD, SKCM, and UVM. For LGG, the expression of *HAVCR2* was positively correlated with B cells, T cells, macrophages, and neutrophils (Figure 5A).

To deeply clarify *HAVCR2* expression and immune infiltration, 24 types of lymphocytes were investigated. The heatmap represented *HAVCR2* expression that was substantially linked with different immune infiltrating cells and their subtypes in pan-cancer (Figure 5B). These findings suggested that many immunocytes were significantly correlated with *HAVCR2* levels. *HAVCR2* gene could play an immune regulation role in CESC, GBM, GBMLGG, KIRC, LGG, PRAD, SKCM, and UVM, which may be associated with its good prognosis. The immune checkpoint pathway performed an essential function to T-cell infiltrating tumors and stopped cancer from growing. Correlations with *HAVCR2* and immune checkpoints near attain the strong level in Figure 5C. Eight prognosis-related cancers, namely, CESC, SKCM, GBM, LGG, GBMLGG, KIRC, PRAD, and UVM, are highly positively correlated with at least 36 of 46 immune checkpoints such as CD200 receptor 1, CD86, and programmed cell death 1 ligand 2 (PCD1LG2). These observed associations existed between *HAVCR2* and recognized immune checkpoints in most cancers, suggesting a potential synergy treatment effect.

The ESTIMATE method was calculated based on immune and stromal scores of cancer tissues to reflect tumor purity. *HAVCR2* expression was closely bound up with tumor purity as there was a strong relation between stromal score, immune score, and ESTIMATE score in all 39 cancers (Figure 5D and Supplementary Figures S1, S2). The data suggested that *HAVCR2* expression might modulate tumor-infiltrating lymphocytes.

### Relevance between *HAVCR2* expression and TMB, MSI, neoantigens, DNA methylation, and genetic alterations

**FIGURE 6:**
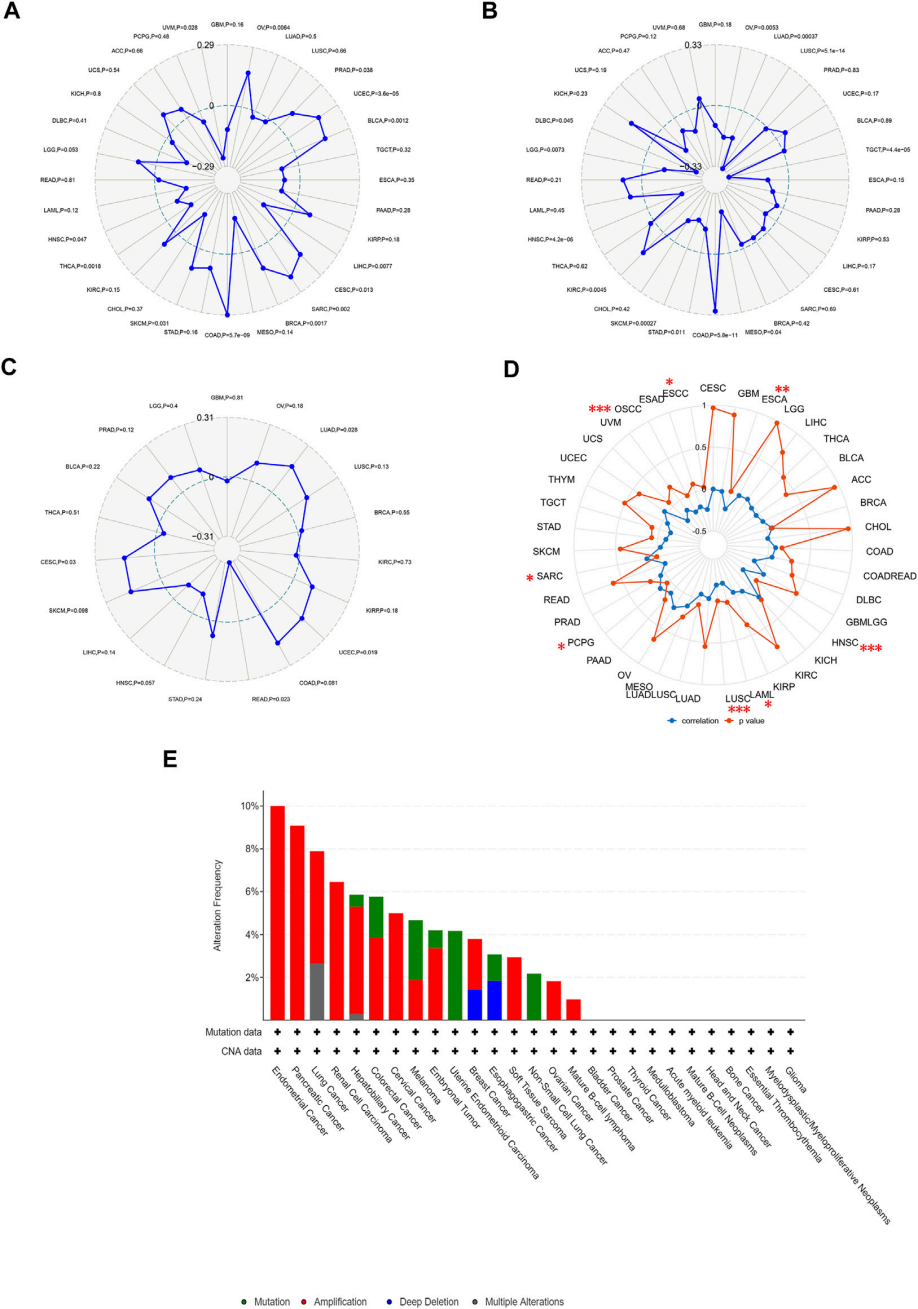
*HAVCR2* gene expression on TMB, MSI, neoantigens, and DNA methylation. **(A)** *HAVCR2* expression was markedly correlated with TMB in CESC (*p* = 0.013), PRAD (*p* = 0.038), SKCM (*p* = 0.031), and UVM (*p* = 0.028) in eight prognosis-related cancers. **(B)** *HAVCR2* expression was conspicuously linked to the MSI of KIRC (*p* = 0.0045), LGG (*p* = 0.0073), and SKCM (*p* = 0.00027) of eight prognosis-related cancers. **(C)** *HAVCR2* expression was notably related to the neoantigens of CESC (*p* = 0.030), LUAD (*p* = 0.028), READ (*p* = 0.023), and UCEC (*p* = 0.019) in eight prognosis-related cancers. **(D)** *HAVCR2* expression linked to DNA methylation in ESCA (*p* = 0.008), ESCC (*p* = 0.035), HNSC (*p* = 4.30E-06), LAML (*p* = 0.035), LUSC (*p* = 2.41E-04), OSCC (*p* = 6.60E-05), PCPG (*p* = 0.046), and SARC (*p* = 0.021) in pan-cancer. **(E)** Alteration frequency of *HAVCR2* with different types of mutations. *p < 0.05, **p < 0.01, and ***p < 0.001.

TMB and MSI are quantitative biomarkers for predicting tumor patients’ response to immunotherapy. It was found that *HAVCR2* gene expression was noticeably related to TMB in SKCM (*p =* 0.031), CESC (*p* = 0.013), PRAD (*p =* 0.038), and UVM (*p =* 0.028) in eight prognosis-related cancers (Figure 6A). We found that the *HAVCR2* gene was strikingly linked to MSI in KIRC (*p =* 0.0045), LGG (*p =* 0.0073), and SKCM (*p =* 0.00027) in eight prognosis-related cancers (Figure 6B). We then evaluated neoantigens in each tumor sample of eight prognosis-related cancers and found CESC (*p =* 0.030), LUAD (*p =* 0.028), READ (*p =* 0.023), and UCEC (*p =* 0.019) were notably related to neoantigens according to the results (Figure 6C). We found *HAVCR2* was conspicuously linked to DNA methylation in ESCA (*p =* 0.008), ESCC (*p =* 0.035), HNSC (*p =* 4.30E-06), LAML (*p =* 0.035), LUSC (*p =* 2.41E-04), OSCC (*p =* 6.60E-05), PCPG (*p =* 0.046), and SARC (*p =* 0.021) (Figure 6D and Table 1). The observed results suggested that *HAVCR2* gene expression had multiple effects on cancer immunity. We further explored the alterations of *HAVCR2* in pan-cancer using the cBioPortal database. The highest alteration frequency of *HAVCR2* was approximately 10%, which appeared for patients with endometrial cancer, and amplification was the most frequent type among different types of genetic alterations (Figure 6E). We also analyzed the mutation patterns of the *HAVCR2* gene in diverse cancers additionally. Mutation rates of *HAVCR2* gene in SKCM, CESC, GBM, KIRC, and PRAD were 2.3, 1.0, 0.2, 0.1, and 0.1%, respectively (Figure 6F and Supplementary Figure S3).

**TABLE 1:** *HAVCR2* expression and DNA methylation in pan-cancer. *p < 0.05, **p < 0.01, and ***p < 0.001.

Characteristic	Correlation	*p*-value
ACC	−0.008	0.941
BLCA	−0.042	0.394
BRCA	0.068	0.057
CESC	0.002	0.969
CHOL	−0.008	0.962
COAD	0.082	0.158
COADREAD	0.053	0.292
DLBC	−0.139	0.344
ESAD	−0.185	0.101
ESCA	−0.210	0.008**
ESCC	−0.235	0.035*
GBM	−0.017	0.905
GBMLGG	0.030	0.482
HNSC	−0.204	<0.001∗∗∗
KICH	0.160	0.202
KIRC	−0.017	0.767
KIRP	−0.055	0.365
LAML	−0.200	0.035∗
LGG	0.001	0.977
LIHC	0.019	0.716
LUAD	−0.028	0.545
LUADLUSC	−0.065	0.063
LUSC	−0.190	<0.001∗∗∗
MESO	0.122	0.263
OSCC	−0.219	<0.001∗∗∗
OV	0.214	0.662
PAAD	0.096	0.203
PCPG	0.150	0.046∗
PRAD	0.058	0.200
READ	−0.052	0.612
SARC	0.143	0.021∗
SKCM	−0.035	0.445
STAD	−0.097	0.075
TGCT	−0.137	0.096
THCA	−0.030	0.501
THYM	−0.062	0.505
UCEC	0.040	0.408
UCS	−0.244	0.070
UVM	−0.145	0.198

### Correlation analysis with DNA repair gene, immune activation, and suppressive genes

**FIGURE 7:**
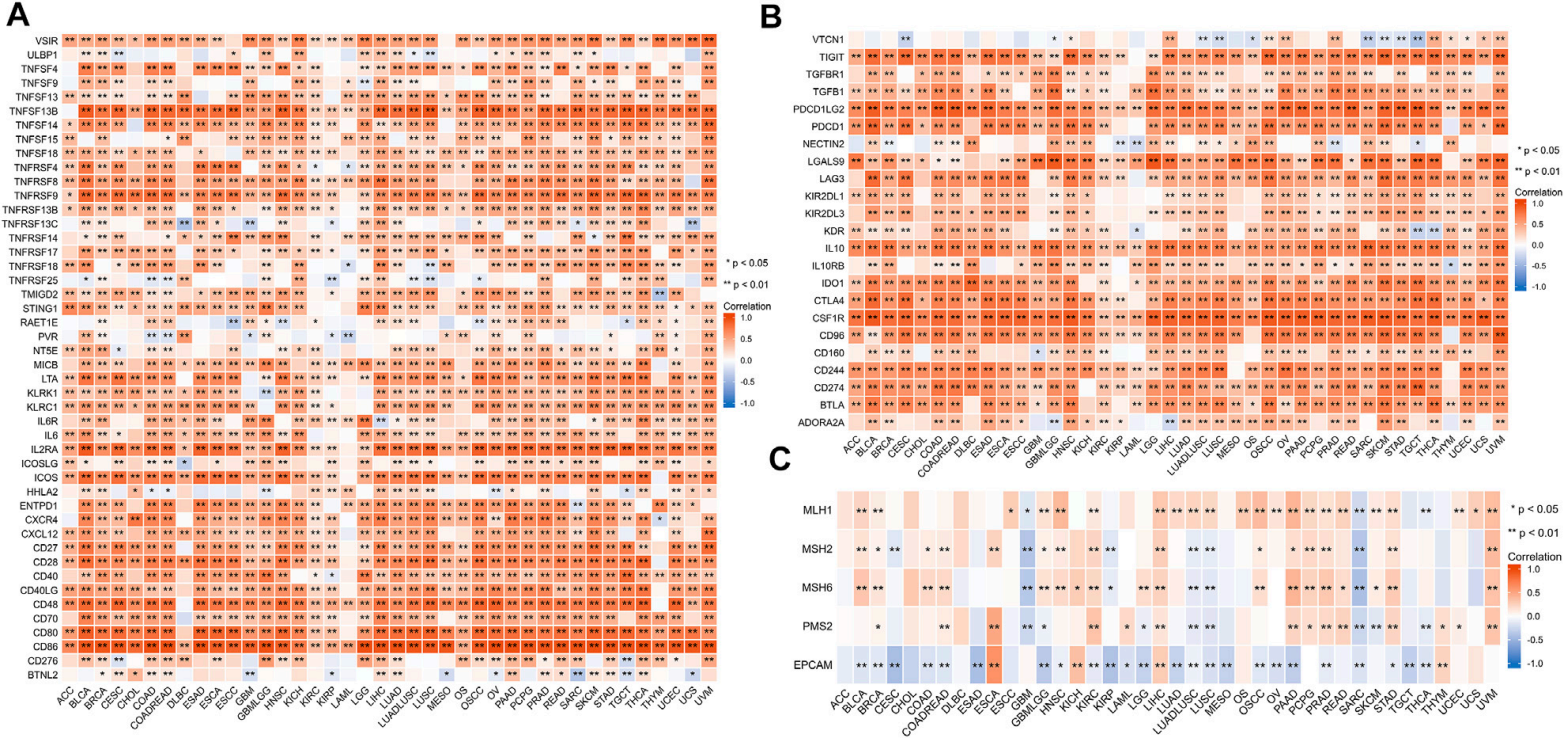
Correlation between *HAVCR2* and immunoregulation-related genes and DNA repair gene in pan-cancer. **(A)** Correlation with *HAVCR2* expression and immune-activating genes. **(B)** Relationship between *HAVCR2* expression and immunosuppressive genes. **(C)** Correlativity of *HAVCR2* expression and DNA repair genes. *p < 0.05, **p < 0.01, and ***p < 0.001.

We investigated the relationship between *HAVCR2* and immunomarker genes related to T-cell functions, such as immune activation and suppressive genes. The resulting heatmap indicated that *HAVCR2* co-expressed with almost all immune activating and suppressive genes positively, such as CD86, PDCD1LG2, interleukin 10 (IL10), and colony-stimulating factor 1 receptor (CSF1R) (Figures 7A,B).

To investigate the association between DNA damage and *HAVCR2* expression, five DNA repair genes (*PMS2*, *MSH2*, *MSH6*, *MLH1*, and *EPCAM* mutations) were used to assess the relationship between *HAVCR2*. Our results indicated different degrees of connection between *HAVCR2* expression and five DNA repair genes in pan-cancer (Figure 7C). All of the aforementioned could reflect the influence of *HAVCR2* in gene-related manners.

### Functional enrichment analysis

**FIGURE 8:**
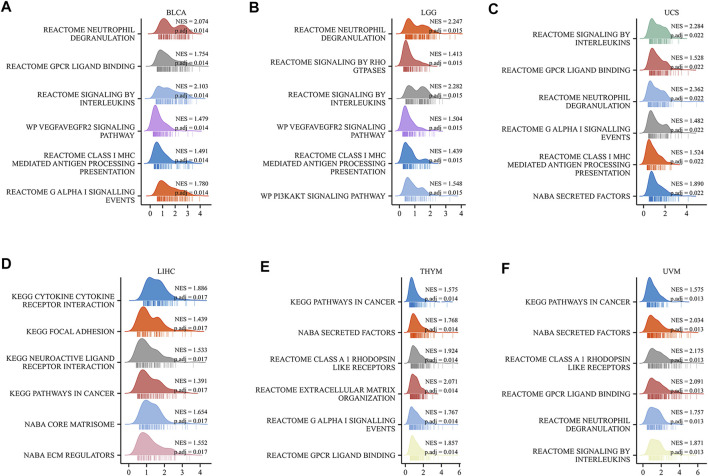
Top six GSEA terms of *HAVCR2* in indicated tumors **(A–F)**.

To study how the *HAVCR2* gene functions biologically, we explored the pathways of *HAVCR2* using GSEA in 39 tumor types from TCGA. Notably, we detected that the *HAVCR2* expression is related to the G protein-coupled receptor (GPCR) ligand binding pathway and interleukin pathway generally, which may regulate tumor metabolism and the microenvironment. Moreover, the result suggested significant enrichment in the immune and tumor terms, including neutrophil degranulation, class I major histocompatibility complex (MHC)-mediated antigen processing presentation, and cancer pathways significantly associated with multiple tumors, such as BLCA, LGG, UCS, LIHC, THYM, and UVM (Figures 8A–F). Other pathways like the cytokine receptor interaction pathway and vascular endothelial growth factor signal pathway are also obviously correlated with *HAVCR2* (Supplementary Figure S4). Altogether, these results suggest that *HAVCR2* expression was a key driver of immune response and cancer.

### Verification of *HAVCR2* expression in pan-cancer

**FIGURE 9:**
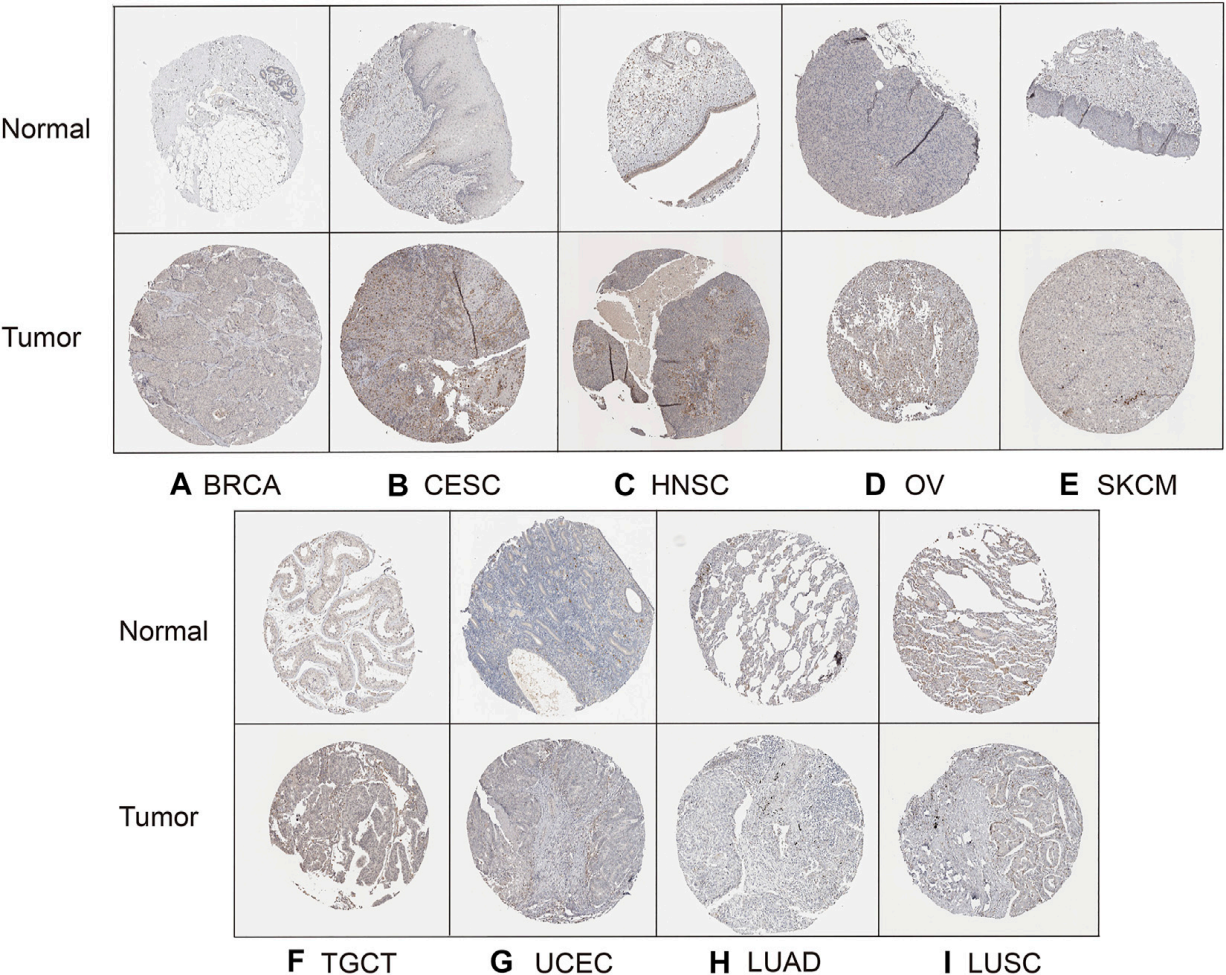
HPA database verified *HAVCR2* gene expression in nine tumors on the protein level. The *HAVCR2* expression in BRCA **(A)**, CESC **(B)**, HNSC **(C)**, OV **(D)**, SKCM **(E)**, TGCT **(F)**, and UCEC **(G)** was significantly upregulated compared to that of corresponding normal tissues. *HAVCR2* gene in LUAD **(H)** and LUSC **(I)** is lower than in normal tissues.

Finally, to evaluate *HAVCR2* expression at the protein level, the HPA database was used. The results confirmed that *HAVCR2* gene expression was remarkably high in BRCA, CESC, HNSC, OV, SKCM, TGCT, and UCEC, consistent with the data from TCGA. Meanwhile, *HAVCR2* gene was expressed low in LUAD and LUSC (Figures 9A–I). Apart from that, we explored *HAVCR2* expression subcellular location through the Sangerbox tool. Location on the cell membrane was essential for the immune checkpoint. Notably, the results showed that *HAVCR2* protein is mainly located on the plasma membrane and in the nucleus and endosomes (Supplementary Figure S5).

## Discussion

Most current cancer therapies focus on killing tumor cells directly, but effectiveness remains limited (Zhao et al., 2021). Oncogenesis is a multistep process consisting of oncogene alteration, genomic instability, epigenetic modifications, tumor microenvironment, abnormal cell signaling, and host immune response (Dawson and Kouzarides, 2012; Jeggo et al., 2016). Prerequisites for improving cancer prognosis are detecting it early and treating effectively. Immune checkpoint blockade therapy has achieved great success by blocking the T-cell function and inducing a lasting anticancer response (Dunn et al., 2002). New studies have reported TIM-3, encoded by *HAVCR2*, as an inhibitory checkpoint protein of tumor-infiltrating T cells. There were a number of T cells infiltrating during tumor progression, but most of them were functionally lost (Jiang et al., 2020). Immune checkpoint proteins maintained a balance between positive and negative signals mediated by T cells and protected tumor cells from immune surveillance in cancers (Kandel et al., 2021). TIM-3 could be expressed on NK cells, dendritic cells, CD8^+^ T cells, monocytes, and other T-cell subsets to regulate cancer immunity (Miller et al., 2019; Solinas et al., 2019).

The present study first demonstrated a comprehensive landscape for *HAVCR2* systematically and extensively to explore its instrumental role in pan-cancer. Our study first provided broad insight into differential expressions and related mechanisms of *HAVCR2* in the pan-cancer dataset. *HAVCR2* exhibited marked upregulation in BRCA, CHOL, CESC, COAD, DLBC, ESCA, LAML, GBM, LIHC, HNSC, KIRC, KIRP, LGG, OV, PAAD, STAD, SKCM, THCA, TGCT, UCS, and UCEC. *HAVCR2* expression correlated with OS, PFI, and DSS in pan-cancer. Downregulation of *HAVCR2* acted as a risk factor in CESC, KIRC, osteosarcoma, and SKCM, while it was protective in GBM, GBMLGG, LGG, PRAD, and UVM. This was consistent with previous reports that TIM-3 was an independent prognostic factor in GBM and PRAD (Liu et al., 2016; Wu et al., 2017). These results indicated that *HAVCR2* could act as a potential predictor for tumor prognosis. A key finding in our study was that *HAVCR2* expression was associated with cancer immunity. We found that *HAVCR2* and infiltration of innate lymphoid cells in multiple cancers were significantly correlated. *HAVCR2* expression is also frequently associated with the majority of common immune markers in CESC, GBM, GBMLGG, KIRC, LGG, PRAD, SKCM, and UVM. Based on these results, our findings revealed that cancer immunity is positively correlated with the *HAVCR2* expression. Each of TMB, MSI, and neoantigens is a strongly correlated predictive factor for the potential immunological efficacy of targeting *HAVCR2*. In line with previously published data, *HAVCR2* expression was closely related to immune infiltration (Das et al., 2017). Tumors of diverse types have been found to express TIM-3, including CD4^+^ and CD8^+^ tumor-infiltrating lymphocytes in BLCA, SKCM, and KIRC. TIM-3 in dendritic cells preferentially interacted with nuclear protein to inhibit nucleic acid recruitment to inner chambers, thereby inhibiting signal transmission of innate immune response (Baitsch et al., 2011; Chiba et al., 2012; Giraldo et al., 2017; Solinas et al., 2017). TIM-3 could also induce Th1 cell apoptosis and promote CD8^+^ T-cell depletion, M2 macrophage polarization, and myeloid-derived suppressor cell proliferation to suppress the immune response. Therefore, TIM-3 facilitated the immunosuppressive tumor microenvironment and led to immune tolerance, thereby promoting tumor occurrence and development (Sheng and Han, 2019). Immune checkpoint inhibitors acting on T cells can restrict the duration and strength of immune responses and maintain tolerance (Baumeister et al., 2016). Tumors can escape from the aforementioned pathways to evade immune eradication. In addition to that, aberrant regulation of antigen and immune-related gene expression might contribute to oncogenesis and immune evasion (Cogdill et al., 2017).

*HAVCR2* expression exerted a pleiotropic effect on malignancy not only regulating immune infiltration but also involving DNA methylation, tumor biology, and metabolism. Hypermethylation often silences or inactivates tumor suppressor genes in cancer (Jones et al., 2019). Our results illuminated that DNA methylation of *HAVCR2* was dysregulated in different cancers. Meanwhile, a strong positive correlation between DNA methylation and *HAVCR2* expression was proved to be related to T-cell activation (McGuire et al., 2019). In all, the specific mechanism between *HAVCR2* expression and DNA methylation warrants more in-depth study.

We studied the biological functions through the enrichment of *HAVCR2* in tumors. The results’ pathways enriched in neutrophil degranulation, class I MHC-mediated antigen processing presentation, and cancer pathway. Our analysis also revealed some other general metabolism and inflammatory-associated pathway terms, such as GPCR ligand binding signaling and interleukin signaling, were also closely associated with *HAVCR2*. As the largest cell membrane receptor family, GPCRs triggered the downstream signaling cascade toward cellular events, and aberrant GPCR activation has been observed in cancer pathogenesis (Luo and Yu, 2019). Interleukins, as important players in large cytokine networks, included key elements that governed tumor immune cell crosstalk and orchestrated the tumor microenvironment (Briukhovetska et al., 2021). Large amounts of Tregs accumulated locally in tumors, and the TIM-3 signal pathway can regulate Tregs immuno-suppressive function by secreting inhibitory cytokines such as transforming growth factor-β and IL10 to promote tumor immune escape (Nishikawa and Sakaguchi, 2010). Consistent with its immunoregulatory actions in cancer, several studies have revealed that TIM-3 also worked in T-cell exhaustion and apoptosis of antigen-specific cytotoxic T lymphocytes in chronic viral infection (Rangachari et al., 2012). These findings could conclude that *HAVCR2* was pivotal in immunity regulation.

Many clinical trials have shown that immunotherapy is effective in cancer treatment. TIM-3 showed drug targets’ potential in our study, and current research studies are exploring modulating or blocking TIM-3 as a therapy for cancer. Targeting TIM-3 had two types of effects including its distinct function by eliminating leukemia stem cells (LSCs) and balancing the immune system indirectly in AML (Kikushige et al., 2010). TIM-3 could selectively kill LSCs but not hematopoietic stem cells (HSCs) in most human AML cells in clinical trials (Kikushige and Akashi, 2012). Based on preclinical data, many trials are exploring the activity of the synergy effect through inhibiting TIM-3 and PD-1 (Hellmann et al., 2021). Some studies provided evidence that PD-1 could interact with TIM-3 and Gal-9 upregulated by inflammatory cytokines to attenuate apoptosis of T cells in cancers (Yang et al., 2021). The levels of PD-1 and TIM-3 protein were significantly correlated in non–small cell lung cancer, suggesting their interplay role in cancers (Datar et al., 2019). The level of TIM-3 is also a potential biomarker for anti-angiogenesis and immunotherapy to evaluate the therapeutic effect (Pignon et al., 2019; Liu et al., 2021).

There are several limitations to our study. Our work is a retrospective study based on public databases. Experiments *in vivo* and *in vitro* should perform follow-up to verify the *HAVCR2*-related pathway on antitumor activity, and additional clinical trials are required to validate treatment efficacy targeting the immune checkpoint of *HAVCR2*.

## Conclusion

In conclusion, differential *HAVCR2* expression was significantly associated with prognosis, immune cell infiltration, and immune-related markers in pan-cancer. Epigenetic changes of *HAVCR2* were observed in many types of cancer. The blockade of immune checkpoint receptors has made great strides in cancer treatment. This study sheds light on the mechanism of *HAVCR2* in tumor immunity and is a promising biomarker for immunotherapy. Future prospective and experimental studies may provide additional perspectives on *HAVCR2* functions in tumors. The *HAVCR2*/TIM-3 pathway represents an intriguing target and could further shape the landscape of cancer immunotherapy.

## Data Availability

The datasets presented in this study can be found in online repositories. The names of the repository/repositories and accession number(s) can be found in the article/Supplementary Material.

## Supplementary material

Supplementary Figure S1
*HAVCR2* gene expression was positively and significantly related to stromal score in all 39 cancers. ****p* < 0.001.

Supplementary Figure S2
*HAVCR2* gene expression was positively and significantly correlated with immune score in all 39 cancers ****p* < 0.001.

Supplementary Figure S3 Mutation rates of *HAVCR2* gene in **(A)** SKCM, **(B)** CESC, **(C)** GBM, **(D)** KIRC, and **(E)** PRAD were 2.3, 1.0, 0.2, 0.1, and 0.1%, respectively.

Supplementary Figure S4 GSEA of *HAVCR2* in other 33 pan-cancers **(A–F)** and top six GSEA terms in indicated tumor types.

Supplementary Figure S5 Subcellular location of *HAVCR2*.

Supplementary Table S1 Baseline characteristic of 39 types of cancer from TCGA.
