# Supplementary material for: Differential expression of HAVCR2 gene in pan-cancer: A potential biomarker for survival and immunotherapy
Source: Front Genet. 2022 Aug 23;13:972664. doi: 10.3389/fgene.2022.972664 (PMC9445440; doi:10.3389/fgene.2022.972664)
Supplement: Supplementary file 1 [file Table1.docx]

| Characteristic | Low expression of HAVCR2 | High expression of HAVCR2 | *p* |
| --- | --- | --- | --- |
| n | 235 | 236 |  |
| Pathologic stage, n (%) |  |  | < 0.001 |
| Stage I | 24 (5.8%) | 53 (12.9%) |  |
| Stage II | 91 (22.1%) | 49 (11.9%) |  |
| Stage III | 81 (19.7%) | 90 (21.8%) |  |
| Stage IV | 11 (2.7%) | 13 (3.2%) |  |
| Gender, n (%) |  |  | 0.731 |
| Female | 87 (18.5%) | 92 (19.5%) |  |
| Male | 148 (31.4%) | 144 (30.6%) |  |
| Age, n (%) |  |  | 0.152 |
| <=60 | 117 (25.3%) | 135 (29.2%) |  |
| >60 | 113 (24.4%) | 98 (21.2%) |  |
| Age, median (IQR) | 60 (49, 72) | 57 (46, 68) | 0.016 |

Supplementary Table 1. Baseline characteristic of 39 types of cancer from TCGA.

SKCM

LIHC

| Characteristic | Low expression of HAVCR2 | High expression of HAVCR2 | *p* |
| --- | --- | --- | --- |
| n | 187 | 187 |  |
| Pathologic stage, n (%) |  |  | 0.811 |
| Stage I | 85 (24.3%) | 88 (25.1%) |  |
| Stage II | 42 (12%) | 45 (12.9%) |  |
| Stage III | 46 (13.1%) | 39 (11.1%) |  |
| Stage IV | 3 (0.9%) | 2 (0.6%) |  |
| Gender, n (%) |  |  | 0.825 |
| Female | 62 (16.6%) | 59 (15.8%) |  |
| Male | 125 (33.4%) | 128 (34.2%) |  |
| Age, n (%) |  |  | 0.277 |
| <=60 | 83 (22.3%) | 94 (25.2%) |  |
| >60 | 104 (27.9%) | 92 (24.7%) |  |
| Age, median (IQR) | 62 (53, 69) | 60 (51, 68) | 0.389 |

BLCA

| Characteristic | Low expression of HAVCR2 | High expression of HAVCR2 | *p* |
| --- | --- | --- | --- |
| n | 207 | 207 |  |
| Pathologic stage, n (%) |  |  | 0.005 |
| Stage I | 3 (0.7%) | 1 (0.2%) |  |
| Stage II | 79 (19.2%) | 51 (12.4%) |  |
| Stage III | 58 (14.1%) | 84 (20.4%) |  |
| Stage IV | 65 (15.8%) | 71 (17.2%) |  |
| Gender, n (%) |  |  | 0.118 |
| Female | 47 (11.4%) | 62 (15%) |  |
| Male | 160 (38.6%) | 145 (35%) |  |
| Age, n (%) |  |  | 0.766 |
| <=70 | 115 (27.8%) | 119 (28.7%) |  |
| >70 | 92 (22.2%) | 88 (21.3%) |  |
| Age, median (IQR) | 69 (59, 77) | 68 (61, 75) | 0.922 |

BRCA

| Characteristic | Low expression of HAVCR2 | High expression of HAVCR2 | *p* |
| --- | --- | --- | --- |
| n | 541 | 542 |  |
| Pathologic stage, n (%) |  |  | 0.779 |
| Stage I | 91 (8.6%) | 90 (8.5%) |  |
| Stage II | 305 (28.8%) | 314 (29.6%) |  |
| Stage III | 123 (11.6%) | 119 (11.2%) |  |
| Stage IV | 11 (1%) | 7 (0.7%) |  |
| Age, n (%) |  |  | 0.484 |
| <=60 | 294 (27.1%) | 307 (28.3%) |  |
| >60 | 247 (22.8%) | 235 (21.7%) |  |
| Age, median (IQR) | 59 (48, 67) | 58 (49, 67) | 0.711 |

CESC

| Characteristic | Low expression of HAVCR2 | High expression of HAVCR2 | *p* |
| --- | --- | --- | --- |
| n | 153 | 153 |  |
| Age, n (%) |  |  | 0.557 |
| <=50 | 97 (31.7%) | 91 (29.7%) |  |
| >50 | 56 (18.3%) | 62 (20.3%) |  |
| Age, median (IQR) | 46 (37, 56) | 46 (39, 58) | 0.404 |

CHOL

| Characteristic | Low expression of HAVCR2 | High expression of HAVCR2 | *p* |
| --- | --- | --- | --- |
| n | 18 | 18 |  |
| Pathologic stage, n (%) |  |  | 1.000 |
| Stage I | 9 (25%) | 10 (27.8%) |  |
| Stage II | 5 (13.9%) | 4 (11.1%) |  |
| Stage III | 0 (0%) | 1 (2.8%) |  |
| Stage IV | 4 (11.1%) | 3 (8.3%) |  |
| Gender, n (%) |  |  | 0.315 |
| Female | 8 (22.2%) | 12 (33.3%) |  |
| Male | 10 (27.8%) | 6 (16.7%) |  |
| Age, n (%) |  |  | 0.505 |
| <=65 | 7 (19.4%) | 10 (27.8%) |  |
| >65 | 11 (30.6%) | 8 (22.2%) |  |
| Age, median (IQR) | 67.5 (58.25, 71.75) | 61 (53.5, 71.75) | 0.438 |

COAD

| Characteristic | Low expression of HAVCR2 | High expression of HAVCR2 | *p* |
| --- | --- | --- | --- |
| n | 239 | 239 |  |
| Pathologic stage, n (%) |  |  | 0.614 |
| Stage I | 43 (9.2%) | 38 (8.1%) |  |
| Stage II | 86 (18.4%) | 101 (21.6%) |  |
| Stage III | 68 (14.6%) | 65 (13.9%) |  |
| Stage IV | 35 (7.5%) | 31 (6.6%) |  |
| Gender, n (%) |  |  | 0.783 |
| Female | 111 (23.2%) | 115 (24.1%) |  |
| Male | 128 (26.8%) | 124 (25.9%) |  |
| Age, n (%) |  |  | 0.514 |
| <=65 | 101 (21.1%) | 93 (19.5%) |  |
| >65 | 138 (28.9%) | 146 (30.5%) |  |
| Age, median (IQR) | 68 (57, 75) | 70 (59.5, 78) | 0.077 |

COADREAD

| Characteristic | Low expression of HAVCR2 | High expression of HAVCR2 | *p* |
| --- | --- | --- | --- |
| n | 322 | 322 |  |
| Pathologic stage, n (%) |  |  | 0.109 |
| Stage I | 62 (10%) | 49 (7.9%) |  |
| Stage II | 104 (16.7%) | 134 (21.5%) |  |
| Stage III | 94 (15.1%) | 90 (14.4%) |  |
| Stage IV | 49 (7.9%) | 41 (6.6%) |  |
| Gender, n (%) |  |  | 0.874 |
| Female | 152 (23.6%) | 149 (23.1%) |  |
| Male | 170 (26.4%) | 173 (26.9%) |  |
| Age, n (%) |  |  | 0.301 |
| <=65 | 145 (22.5%) | 131 (20.3%) |  |
| >65 | 177 (27.5%) | 191 (29.7%) |  |
| Age, median (IQR) | 66.5 (57, 74) | 69 (59, 77) | 0.037 |

DLBC

| Characteristic | Low expression of HAVCR2 | High expression of HAVCR2 | *p* |
| --- | --- | --- | --- |
| n | 24 | 24 |  |
| Gender, n (%) |  |  | 1.000 |
| Female | 13 (27.1%) | 13 (27.1%) |  |
| Male | 11 (22.9%) | 11 (22.9%) |  |
| Age, n (%) |  |  | 0.561 |
| <=60 | 15 (31.2%) | 12 (25%) |  |
| >60 | 9 (18.8%) | 12 (25%) |  |
| Age, mean ± SD | 56.46 ± 12.8 | 56.08 ± 15.28 | 0.927 |

ESAD

| Characteristic | Low expression of HAVCR2 | High expression of HAVCR2 | *p* |
| --- | --- | --- | --- |
| n | 40 | 40 |  |
| Pathologic stage, n (%) |  |  | 0.338 |
| Stage I | 7 (11.1%) | 2 (3.2%) |  |
| Stage II | 10 (15.9%) | 12 (19%) |  |
| Stage III | 12 (19%) | 15 (23.8%) |  |
| Stage IV | 2 (3.2%) | 3 (4.8%) |  |
| Gender, n (%) |  |  | 1.000 |
| Female | 5 (6.2%) | 6 (7.5%) |  |
| Male | 35 (43.8%) | 34 (42.5%) |  |
| Age, n (%) |  |  | 0.248 |
| <=60 | 18 (22.5%) | 12 (15%) |  |
| >60 | 22 (27.5%) | 28 (35%) |  |
| Age, median (IQR) | 63 (56.75, 71.25) | 74 (60, 77) | 0.030 |

ESCA

| Characteristic | Low expression of HAVCR2 | High expression of HAVCR2 | *p* |
| --- | --- | --- | --- |
| n | 81 | 81 |  |
| Pathologic stage, n (%) |  |  | 0.157 |
| Stage I | 12 (8.5%) | 4 (2.8%) |  |
| Stage II | 32 (22.5%) | 37 (26.1%) |  |
| Stage III | 22 (15.5%) | 27 (19%) |  |
| Stage IV | 3 (2.1%) | 5 (3.5%) |  |
| Gender, n (%) |  |  | 0.653 |
| Female | 13 (8%) | 10 (6.2%) |  |
| Male | 68 (42%) | 71 (43.8%) |  |
| Age, n (%) |  |  | 0.530 |
| <=60 | 44 (27.2%) | 39 (24.1%) |  |
| >60 | 37 (22.8%) | 42 (25.9%) |  |
| Age, median (IQR) | 59 (54, 69) | 61 (53, 75) | 0.269 |

ESCC

| Characteristic | Low expression of HAVCR2 | High expression of HAVCR2 | *p* |
| --- | --- | --- | --- |
| n | 41 | 41 |  |
| Pathologic stage, n (%) |  |  | 0.634 |
| Stage I | 5 (6.3%) | 2 (2.5%) |  |
| Stage II | 22 (27.8%) | 25 (31.6%) |  |
| Stage III | 10 (12.7%) | 12 (15.2%) |  |
| Stage IV | 1 (1.3%) | 2 (2.5%) |  |
| Gender, n (%) |  |  | 0.349 |
| Female | 8 (9.8%) | 4 (4.9%) |  |
| Male | 33 (40.2%) | 37 (45.1%) |  |
| Age, n (%) |  |  | 1.000 |
| <=60 | 26 (31.7%) | 27 (32.9%) |  |
| >60 | 15 (18.3%) | 14 (17.1%) |  |
| Age, mean ± SD | 58.44 ± 9.92 | 58.12 ± 11.04 | 0.892 |

GBM

| Characteristic | Low expression of HAVCR2 | High expression of HAVCR2 | *p* |
| --- | --- | --- | --- |
| n | 84 | 84 |  |
| Gender, n (%) |  |  | 0.106 |
| Female | 35 (20.8%) | 24 (14.3%) |  |
| Male | 49 (29.2%) | 60 (35.7%) |  |
| Age, n (%) |  |  | 1.000 |
| <=60 | 43 (25.6%) | 44 (26.2%) |  |
| >60 | 41 (24.4%) | 40 (23.8%) |  |
| Age, mean ± SD | 59.49 ± 13.44 | 58.95 ± 13.68 | 0.798 |

GBMLGG

| Characteristic | Low expression of HAVCR2 | High expression of HAVCR2 | *p* |
| --- | --- | --- | --- |
| n | 348 | 348 |  |
| Gender, n (%) |  |  | 0.146 |
| Female | 159 (22.8%) | 139 (20%) |  |
| Male | 189 (27.2%) | 209 (30%) |  |
| Age, n (%) |  |  | 0.009 |
| <=60 | 291 (41.8%) | 262 (37.6%) |  |
| >60 | 57 (8.2%) | 86 (12.4%) |  |
| Age, median (IQR) | 43 (34, 55.25) | 49 (35, 60) | 0.002 |

HNSC

| Characteristic | Low expression of HAVCR2 | High expression of HAVCR2 | *p* |
| --- | --- | --- | --- |
| n | 251 | 251 |  |
| Gender, n (%) |  |  | 0.130 |
| Female | 59 (11.8%) | 75 (14.9%) |  |
| Male | 192 (38.2%) | 176 (35.1%) |  |
| Age, median (IQR) | 60 (53, 68) | 61 (54, 69) | 0.268 |

KICH

| Characteristic | Low expression of HAVCR2 | High expression of HAVCR2 | *p* |
| --- | --- | --- | --- |
| n | 32 | 33 |  |
| Pathologic stage, n (%) |  |  | 0.169 |
| Stage I | 7 (10.8%) | 13 (20%) |  |
| Stage II | 13 (20%) | 12 (18.5%) |  |
| Stage III | 10 (15.4%) | 4 (6.2%) |  |
| Stage IV | 2 (3.1%) | 4 (6.2%) |  |
| Gender, n (%) |  |  | 0.510 |
| Female | 11 (16.9%) | 15 (23.1%) |  |
| Male | 21 (32.3%) | 18 (27.7%) |  |
| Age, n (%) |  |  | 0.711 |
| <=50 | 15 (23.1%) | 18 (27.7%) |  |
| >50 | 17 (26.2%) | 15 (23.1%) |  |
| Age, mean ± SD | 51.53 ± 14.17 | 52.21 ± 14.24 | 0.847 |

KIRC

| Characteristic | Low expression of HAVCR2 | High expression of HAVCR2 | *p* |
| --- | --- | --- | --- |
| n | 269 | 270 |  |
| Pathologic stage, n (%) |  |  | 0.438 |
| Stage I | 129 (24.1%) | 143 (26.7%) |  |
| Stage II | 32 (6%) | 27 (5%) |  |
| Stage III | 68 (12.7%) | 55 (10.3%) |  |
| Stage IV | 39 (7.3%) | 43 (8%) |  |
| Gender, n (%) |  |  | 0.025 |
| Female | 80 (14.8%) | 106 (19.7%) |  |
| Male | 189 (35.1%) | 164 (30.4%) |  |
| Age, n (%) |  |  | 0.245 |
| <=60 | 127 (23.6%) | 142 (26.3%) |  |
| >60 | 142 (26.3%) | 128 (23.7%) |  |
| Age, mean ± SD | 61.3 ± 11.95 | 59.96 ± 12.21 | 0.199 |

KIRP

| Characteristic | Low expression of HAVCR2 | High expression of HAVCR2 | *p* |
| --- | --- | --- | --- |
| n | 144 | 145 |  |
| Gender, n (%) |  |  | 0.271 |
| Female | 43 (14.9%) | 34 (11.8%) |  |
| Male | 101 (34.9%) | 111 (38.4%) |  |
| Age, n (%) |  |  | 0.813 |
| <=60 | 65 (22.7%) | 68 (23.8%) |  |
| >60 | 78 (27.3%) | 75 (26.2%) |  |
| Pathologic stage, n (%) |  |  | 0.294 |
| Stage I | 82 (31.5%) | 90 (34.6%) |  |
| Stage II | 8 (3.1%) | 14 (5.4%) |  |
| Stage III | 27 (10.4%) | 24 (9.2%) |  |
| Stage IV | 10 (3.8%) | 5 (1.9%) |  |
| Age, median (IQR) | 62 (54, 71.5) | 61 (53, 69) | 0.477 |

LAML

| Characteristic | Low expression of HAVCR2 | High expression of HAVCR2 | *p* |
| --- | --- | --- | --- |
| n | 75 | 76 |  |
| Gender, n (%) |  |  | 0.928 |
| Female | 33 (21.9%) | 35 (23.2%) |  |
| Male | 42 (27.8%) | 41 (27.2%) |  |
| Age, n (%) |  |  | 0.690 |
| <=60 | 42 (27.8%) | 46 (30.5%) |  |
| >60 | 33 (21.9%) | 30 (19.9%) |  |
| Age, median (IQR) | 58 (43.5, 67) | 55 (41.75, 66.25) | 0.374 |

LGG

| Characteristic | Low expression of HAVCR2 | High expression of HAVCR2 | *p* |
| --- | --- | --- | --- |
| n | 264 | 264 |  |
| Gender, n (%) |  |  | 0.861 |
| Female | 118 (22.3%) | 121 (22.9%) |  |
| Male | 146 (27.7%) | 143 (27.1%) |  |
| Age, n (%) |  |  | 0.030 |
| <=40 | 119 (22.5%) | 145 (27.5%) |  |
| >40 | 145 (27.5%) | 119 (22.5%) |  |
| Age, median (IQR) | 42 (33, 53.25) | 38.5 (31, 52) | 0.073 |

LUAD

| Characteristic | Low expression of HAVCR2 | High expression of HAVCR2 | *p* |
| --- | --- | --- | --- |
| n | 267 | 268 |  |
| Pathologic stage, n (%) |  |  | 0.329 |
| Stage I | 144 (27.3%) | 150 (28.5%) |  |
| Stage II | 56 (10.6%) | 67 (12.7%) |  |
| Stage III | 48 (9.1%) | 36 (6.8%) |  |
| Stage IV | 15 (2.8%) | 11 (2.1%) |  |
| Gender, n (%) |  |  | 0.109 |
| Female | 133 (24.9%) | 153 (28.6%) |  |
| Male | 134 (25%) | 115 (21.5%) |  |
| Age, n (%) |  |  | 0.078 |
| <=65 | 138 (26.7%) | 117 (22.7%) |  |
| >65 | 120 (23.3%) | 141 (27.3%) |  |
| Age, median (IQR) | 64.5 (58, 71.75) | 67 (59, 73) | 0.022 |

LUADLUSC

| Characteristic | Low expression of HAVCR2 | High expression of HAVCR2 | *p* |
| --- | --- | --- | --- |
| n | 518 | 519 |  |
| Pathologic stage, n (%) |  |  | 0.484 |
| Stage I | 259 (25.3%) | 280 (27.3%) |  |
| Stage II | 150 (14.6%) | 135 (13.2%) |  |
| Stage III | 86 (8.4%) | 82 (8%) |  |
| Stage IV | 19 (1.9%) | 14 (1.4%) |  |
| Gender, n (%) |  |  | < 0.001 |
| Female | 167 (16.1%) | 250 (24.1%) |  |
| Male | 351 (33.8%) | 269 (25.9%) |  |
| Age, n (%) |  |  | 0.218 |
| <=65 | 233 (23.1%) | 213 (21.1%) |  |
| >65 | 271 (26.9%) | 292 (28.9%) |  |
| Age, median (IQR) | 67 (60, 72) | 68 (60, 74) | 0.036 |

LUSC

| Characteristic | Low expression of HAVCR2 | High expression of HAVCR2 | *p* |
| --- | --- | --- | --- |
| n | 251 | 251 |  |
| Pathologic stage, n (%) |  |  | 0.375 |
| Stage I | 114 (22.9%) | 131 (26.3%) |  |
| Stage II | 89 (17.9%) | 73 (14.7%) |  |
| Stage III | 44 (8.8%) | 40 (8%) |  |
| Stage IV | 3 (0.6%) | 4 (0.8%) |  |
| Gender, n (%) |  |  | < 0.001 |
| Female | 46 (9.2%) | 85 (16.9%) |  |
| Male | 205 (40.8%) | 166 (33.1%) |  |
| Age, n (%) |  |  | 0.793 |
| <=65 | 93 (18.9%) | 98 (19.9%) |  |
| >65 | 152 (30.8%) | 150 (30.4%) |  |
| Age, median (IQR) | 68 (61, 73) | 68 (62, 74) | 0.603 |

MESO

| Characteristic | Low expression of HAVCR2 | High expression of HAVCR2 | *p* |
| --- | --- | --- | --- |
| n | 43 | 43 |  |
| Pathologic stage, n (%) |  |  | 0.448 |
| Stage I | 5 (5.8%) | 5 (5.8%) |  |
| Stage II | 9 (10.5%) | 7 (8.1%) |  |
| Stage III | 24 (27.9%) | 20 (23.3%) |  |
| Stage IV | 5 (5.8%) | 11 (12.8%) |  |
| Gender, n (%) |  |  | 0.570 |
| Female | 9 (10.5%) | 6 (7%) |  |
| Male | 34 (39.5%) | 37 (43%) |  |
| Age, n (%) |  |  | 0.665 |
| <=65 | 22 (25.6%) | 25 (29.1%) |  |
| >65 | 21 (24.4%) | 18 (20.9%) |  |
| Age, median (IQR) | 65 (56, 71) | 62 (57.5, 67.5) | 0.604 |

OV

| Characteristic | Low expression of HAVCR2 | High expression of HAVCR2 | *p* |
| --- | --- | --- | --- |
| n | 189 | 190 |  |
| Age, n (%) |  |  | 0.505 |
| <=60 | 100 (26.4%) | 108 (28.5%) |  |
| >60 | 89 (23.5%) | 82 (21.6%) |  |
| Age, median (IQR) | 59 (51, 69) | 58 (50.25, 67) | 0.546 |

PAAD

| Characteristic | Low expression of HAVCR2 | High expression of HAVCR2 | *p* |
| --- | --- | --- | --- |
| n | 89 | 89 |  |
| Pathologic stage, n (%) |  |  | 0.509 |
| Stage I | 13 (7.4%) | 8 (4.6%) |  |
| Stage II | 68 (38.9%) | 78 (44.6%) |  |
| Stage III | 2 (1.1%) | 1 (0.6%) |  |
| Stage IV | 3 (1.7%) | 2 (1.1%) |  |
| Gender, n (%) |  |  | 0.292 |
| Female | 36 (20.2%) | 44 (24.7%) |  |
| Male | 53 (29.8%) | 45 (25.3%) |  |
| Age, n (%) |  |  | 1.000 |
| <=65 | 47 (26.4%) | 46 (25.8%) |  |
| >65 | 42 (23.6%) | 43 (24.2%) |  |
| Age, mean ± SD | 64.9 ± 10.78 | 64.6 ± 10.88 | 0.852 |

PCPG

| Characteristic | Low expression of HAVCR2 | High expression of HAVCR2 | *p* |
| --- | --- | --- | --- |
| n | 91 | 92 |  |
| Gender, n (%) |  |  | 0.155 |
| Female | 56 (30.6%) | 46 (25.1%) |  |
| Male | 35 (19.1%) | 46 (25.1%) |  |
| Age, n (%) |  |  | 0.507 |
| <=50 | 51 (27.9%) | 57 (31.1%) |  |
| >50 | 40 (21.9%) | 35 (19.1%) |  |
| Age, median (IQR) | 46 (35, 60) | 46 (36.75, 57) | 1.000 |

PRAD

| Characteristic | Low expression of HAVCR2 | High expression of HAVCR2 | *p* |
| --- | --- | --- | --- |
| n | 249 | 250 |  |
| Age, n (%) |  |  | 0.008 |
| <=60 | 127 (25.5%) | 97 (19.4%) |  |
| >60 | 122 (24.4%) | 153 (30.7%) |  |
| Age, median (IQR) | 60 (56, 65) | 63 (57, 66) | 0.006 |

READ

| Characteristic | Low expression of HAVCR2 | High expression of HAVCR2 | *p* |
| --- | --- | --- | --- |
| n | 91 | 92 |  |
| Gender, n (%) |  |  | 0.085 |
| Female | 57 (31.1%) | 45 (24.6%) |  |
| Male | 34 (18.6%) | 47 (25.7%) |  |
| Age, n (%) |  |  | 0.335 |
| <=50 | 50 (27.3%) | 58 (31.7%) |  |
| >50 | 41 (22.4%) | 34 (18.6%) |  |
| Age, median (IQR) | 45 (35, 61) | 46 (40, 56.25) | 0.835 |

SARC

| Characteristic | Low expression of HAVCR2 | High expression of HAVCR2 | *p* |
| --- | --- | --- | --- |
| n | 83 | 83 |  |
| Pathologic stage, n (%) |  |  | 0.518 |
| Stage I | 17 (10.9%) | 13 (8.3%) |  |
| Stage II | 21 (13.5%) | 30 (19.2%) |  |
| Stage III | 27 (17.3%) | 24 (15.4%) |  |
| Stage IV | 12 (7.7%) | 12 (7.7%) |  |
| Gender, n (%) |  |  | 0.061 |
| Female | 44 (26.5%) | 31 (18.7%) |  |
| Male | 39 (23.5%) | 52 (31.3%) |  |
| Age, n (%) |  |  | 0.877 |
| <=65 | 40 (24.1%) | 42 (25.3%) |  |
| >65 | 43 (25.9%) | 41 (24.7%) |  |
| Age, mean ± SD | 64.22 ± 12.79 | 64.92 ± 10.52 | 0.701 |

STAD

| Characteristic | Low expression of HAVCR2 | High expression of HAVCR2 | *p* |
| --- | --- | --- | --- |
| n | 187 | 188 |  |
| Pathologic stage, n (%) |  |  | 0.200 |
| Stage I | 33 (9.4%) | 20 (5.7%) |  |
| Stage II | 54 (15.3%) | 57 (16.2%) |  |
| Stage III | 69 (19.6%) | 81 (23%) |  |
| Stage IV | 21 (6%) | 17 (4.8%) |  |
| Gender, n (%) |  |  | 0.884 |
| Female | 68 (18.1%) | 66 (17.6%) |  |
| Male | 119 (31.7%) | 122 (32.5%) |  |
| Age, n (%) |  |  | 0.106 |
| <=65 | 90 (24.3%) | 74 (19.9%) |  |
| >65 | 95 (25.6%) | 112 (30.2%) |  |
| Age, median (IQR) | 66 (58, 72) | 69 (59, 75) | 0.035 |

TGCT

| Characteristic | Low expression of HAVCR2 | High expression of HAVCR2 | *p* |
| --- | --- | --- | --- |
| n | 69 | 70 |  |
| Pathologic stage, n (%) |  |  | 0.042 |
| Stage I | 53 (40.2%) | 53 (40.2%) |  |
| Stage II | 2 (1.5%) | 10 (7.6%) |  |
| Stage III | 9 (6.8%) | 5 (3.8%) |  |
| Age, n (%) |  |  | 0.674 |
| <=30 | 35 (25.2%) | 32 (23%) |  |
| >30 | 34 (24.5%) | 38 (27.3%) |  |
| Age, median (IQR) | 30 (24, 38) | 31 (27, 36.75) | 0.377 |

THCA

| Characteristic | Low expression of HAVCR2 | High expression of HAVCR2 | *p* |
| --- | --- | --- | --- |
| n | 255 | 255 |  |
| Pathologic stage, n (%) |  |  | 0.114 |
| Stage I | 135 (26.6%) | 151 (29.7%) |  |
| Stage II | 33 (6.5%) | 19 (3.7%) |  |
| Stage III | 53 (10.4%) | 60 (11.8%) |  |
| Stage IV | 32 (6.3%) | 25 (4.9%) |  |
| Gender, n (%) |  |  | 0.842 |
| Female | 184 (36.1%) | 187 (36.7%) |  |
| Male | 71 (13.9%) | 68 (13.3%) |  |
| Age, n (%) |  |  | 0.156 |
| <=45 | 112 (22%) | 129 (25.3%) |  |
| >45 | 143 (28%) | 126 (24.7%) |  |
| Age, median (IQR) | 48 (35, 60.5) | 45 (34.5, 56) | 0.263 |

THYM

| Characteristic | Low expression of HAVCR2 | High expression of HAVCR2 | *p* |
| --- | --- | --- | --- |
| n | 59 | 60 |  |
| Gender, n (%) |  |  | 0.780 |
| Female | 27 (22.7%) | 30 (25.2%) |  |
| Male | 32 (26.9%) | 30 (25.2%) |  |
| Age, n (%) |  |  | 0.027 |
| <=60 | 37 (31.4%) | 24 (20.3%) |  |
| >60 | 22 (18.6%) | 35 (29.7%) |  |
| Age, median (IQR) | 54 (45.5, 64) | 63 (50.5, 70) | 0.056 |

UCEC

| Characteristic | Low expression of HAVCR2 | High expression of HAVCR2 | *p* |
| --- | --- | --- | --- |
| n | 276 | 276 |  |
| Age, n (%) |  |  | 0.716 |
| <=60 | 101 (18.4%) | 105 (19.1%) |  |
| >60 | 175 (31.9%) | 168 (30.6%) |  |
| Age, median (IQR) | 64 (57, 71.25) | 63 (57, 71) | 0.267 |

UCS

| Characteristic | Low expression of HAVCR2 | High expression of HAVCR2 | *p* |
| --- | --- | --- | --- |
| n | 28 | 28 |  |
| Age, n (%) |  |  | 1.000 |
| <=65 | 11 (19.6%) | 11 (19.6%) |  |
| >65 | 17 (30.4%) | 17 (30.4%) |  |
| Age, mean ± SD | 69.93 ± 8.73 | 69.61 ± 10.15 | 0.899 |

UVM

| Characteristic | Low expression of HAVCR2 | High expression of HAVCR2 | *p* |
| --- | --- | --- | --- |
| n | 40 | 40 |  |
| Pathologic stage, n (%) |  |  | 0.864 |
| Stage II | 21 (26.6%) | 18 (22.8%) |  |
| Stage III | 17 (21.5%) | 19 (24.1%) |  |
| Stage IV | 2 (2.5%) | 2 (2.5%) |  |
| Age, n (%) |  |  | 0.502 |
| <=60 | 18 (22.5%) | 22 (27.5%) |  |
| >60 | 22 (27.5%) | 18 (22.5%) |  |
| Gender, n (%) |  |  | 1.000 |
| Female | 18 (22.5%) | 17 (21.2%) |  |
| Male | 22 (27.5%) | 23 (28.7%) |  |
| Age, mean ± SD | 61.55 ± 13.61 | 61.75 ± 14.44 | 0.949 |

ESCA

| Characteristic | Low expression of HAVCR2 | High expression of HAVCR2 | *p* |
| --- | --- | --- | --- |
| n | 81 | 81 |  |
| Pathologic stage, n (%) |  |  | 0.157 |
| Stage I | 12 (8.5%) | 4 (2.8%) |  |
| Stage II | 32 (22.5%) | 37 (26.1%) |  |
| Stage III | 22 (15.5%) | 27 (19%) |  |
| Stage IV | 3 (2.1%) | 5 (3.5%) |  |
| Gender, n (%) |  |  | 0.653 |
| Female | 13 (8%) | 10 (6.2%) |  |
| Male | 68 (42%) | 71 (43.8%) |  |
| Age, n (%) |  |  | 0.530 |
| <=60 | 44 (27.2%) | 39 (24.1%) |  |
| >60 | 37 (22.8%) | 42 (25.9%) |  |
| Age, median (IQR) | 59 (54, 69) | 61 (53, 75) | 0.269 |

ACC

| Characteristic | Low expression of hsa-let-7a-5p | High expression of hsa-let-7a-5p | *p* |
| --- | --- | --- | --- |
| n | 40 | 40 |  |
| Pathologic stage, n (%) |  |  | 0.755 |
| Stage I | 5 (6.4%) | 4 (5.1%) |  |
| Stage II | 18 (23.1%) | 19 (24.4%) |  |
| Stage III | 7 (9%) | 9 (11.5%) |  |
| Stage IV | 10 (12.8%) | 6 (7.7%) |  |
| Gender, n (%) |  |  | 1.000 |
| Female | 25 (31.2%) | 24 (30%) |  |
| Male | 15 (18.8%) | 16 (20%) |  |
| Age, n (%) |  |  | 1.000 |
| <=50 | 21 (26.2%) | 21 (26.2%) |  |
| >50 | 19 (23.8%) | 19 (23.8%) |  |
| Age, mean ± SD | 46.85 ± 15.82 | 45.95 ± 16.15 | 0.802 |

OSCC

| Characteristic | Low expression of HAVCR2 | High expression of HAVCR2 | *p* |
| --- | --- | --- | --- |
| n | 164 | 165 |  |
| Gender, n (%) |  |  | 0.203 |
| Female | 45 (13.7%) | 57 (17.3%) |  |
| Male | 119 (36.2%) | 108 (32.8%) |  |
| Age, n (%) |  |  | 0.376 |
| <=60 | 82 (25%) | 73 (22.3%) |  |
| >60 | 82 (25%) | 91 (27.7%) |  |
| Age, median (IQR) | 60.5 (52, 69) | 62 (55, 73) | 0.138 |
