# Supplementary figures and images for: Differential expression of HAVCR2 gene in pan-cancer: A potential biomarker for survival and immunotherapy
Source: Front Genet. 2022 Aug 23;13:972664. doi: 10.3389/fgene.2022.972664 (PMC9445440; doi:10.3389/fgene.2022.972664)

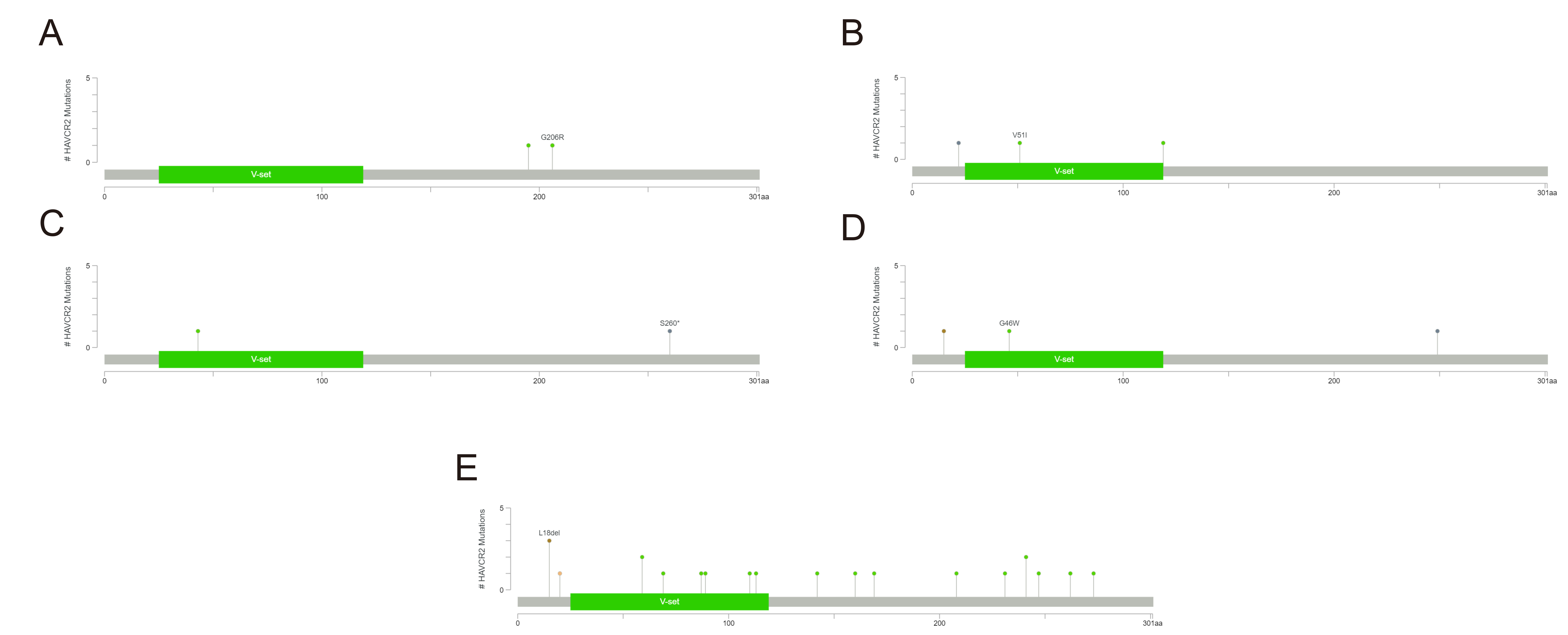

Supplement: Supplementary file 2 [file Image3.TIF]

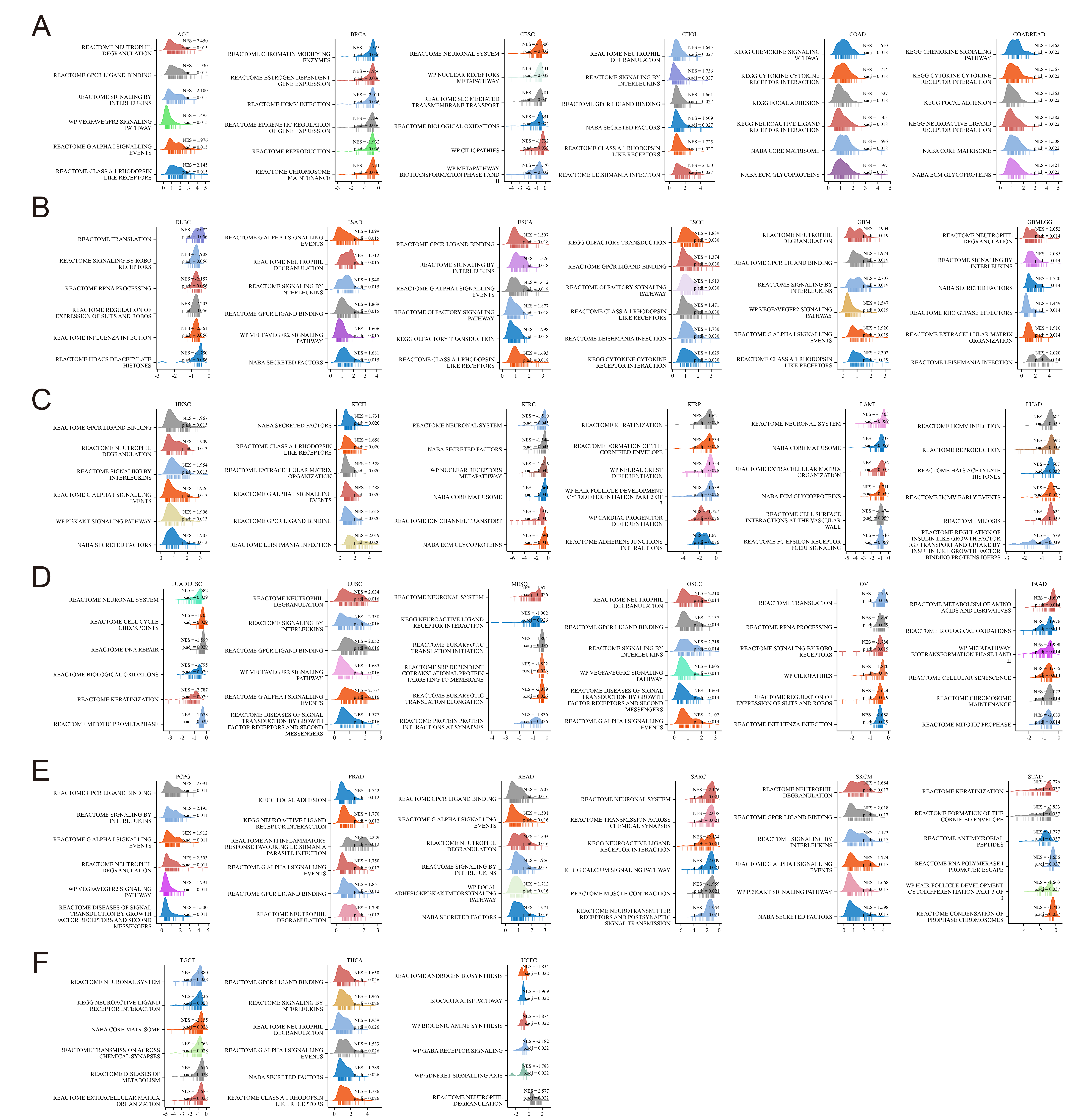

Supplement: Supplementary file 3 [file Image4.TIF]

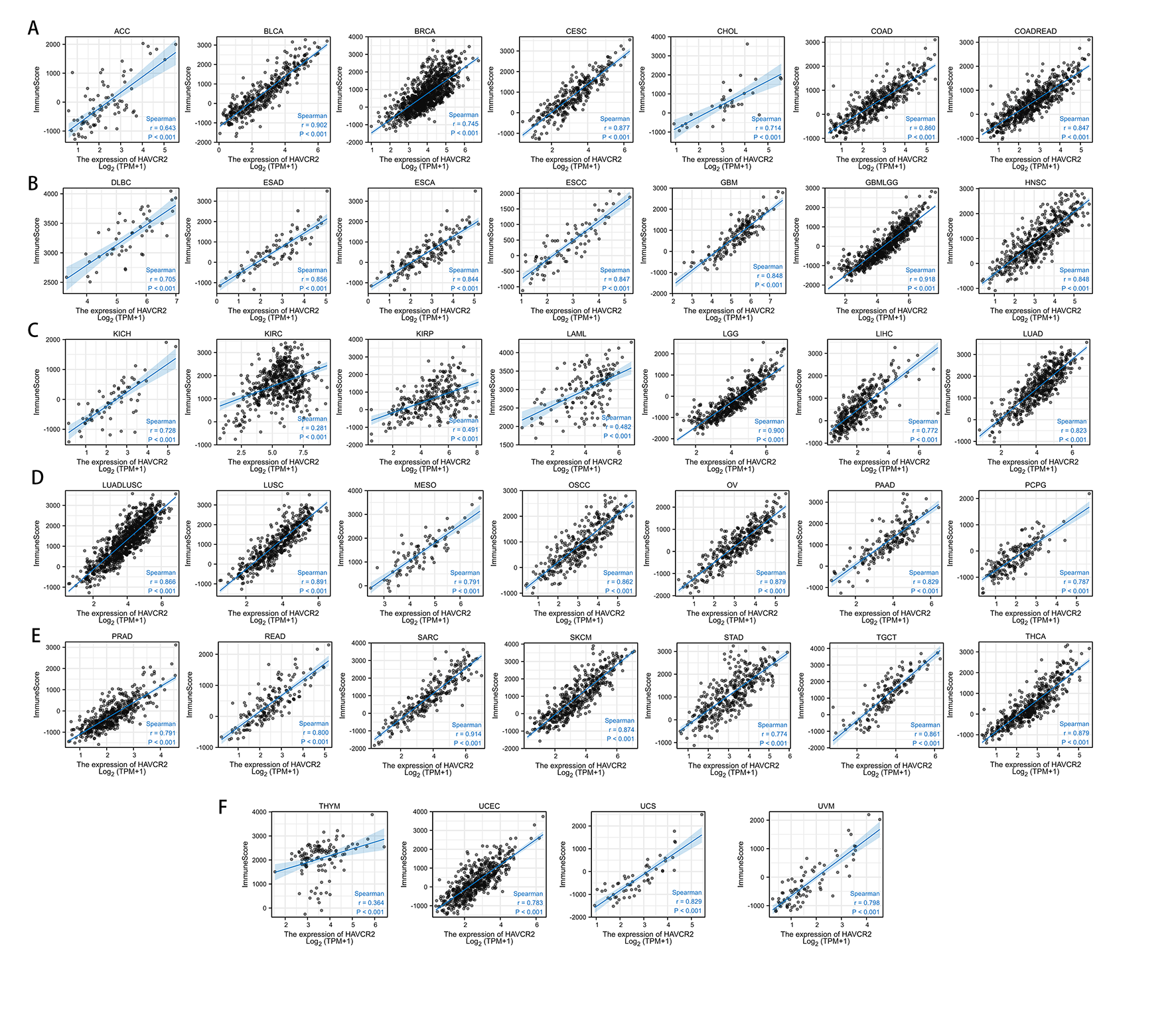

Supplement: Supplementary file 4 [file Image2.TIF]

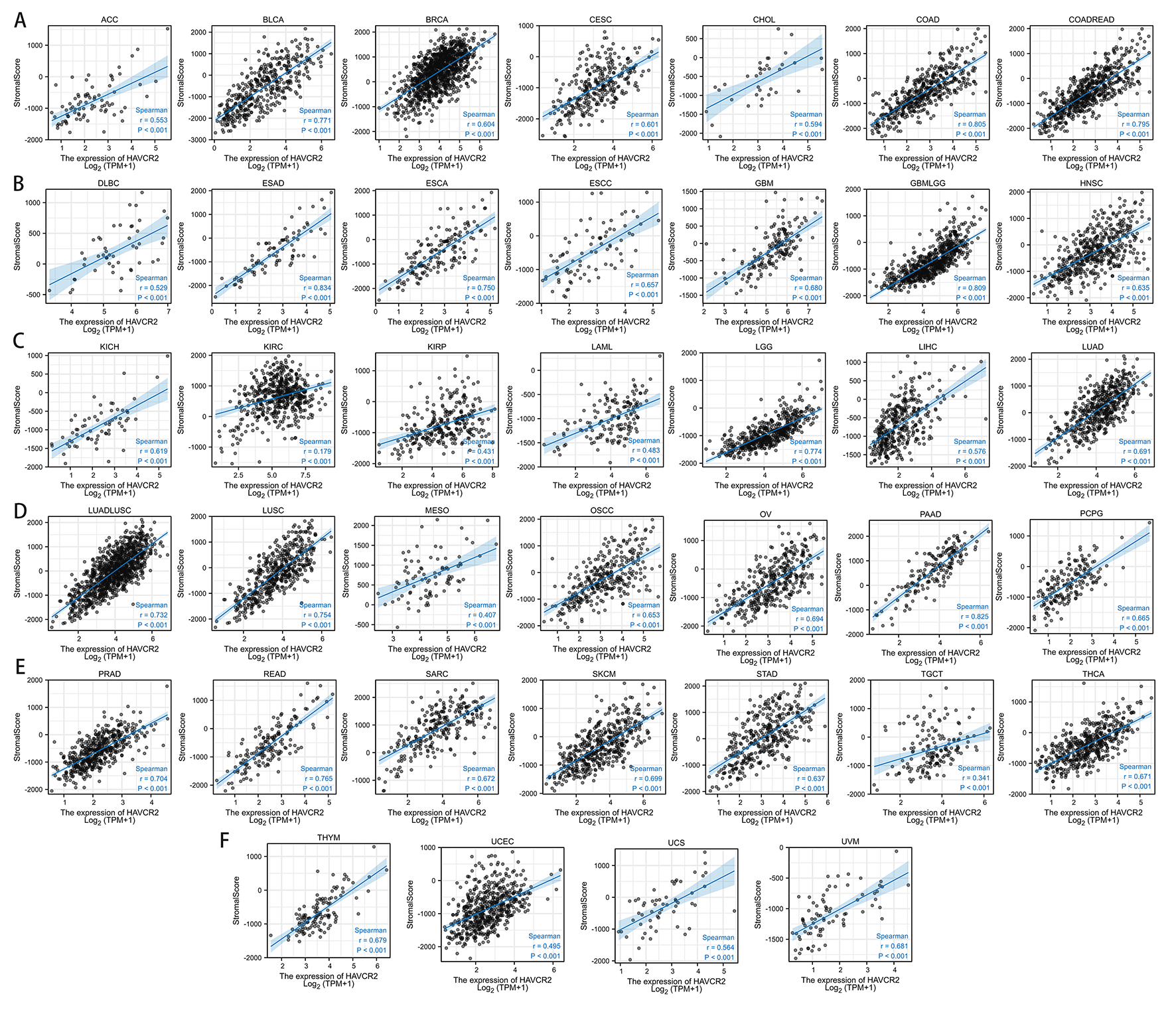

Supplement: Supplementary file 5 [file Image1.TIF]

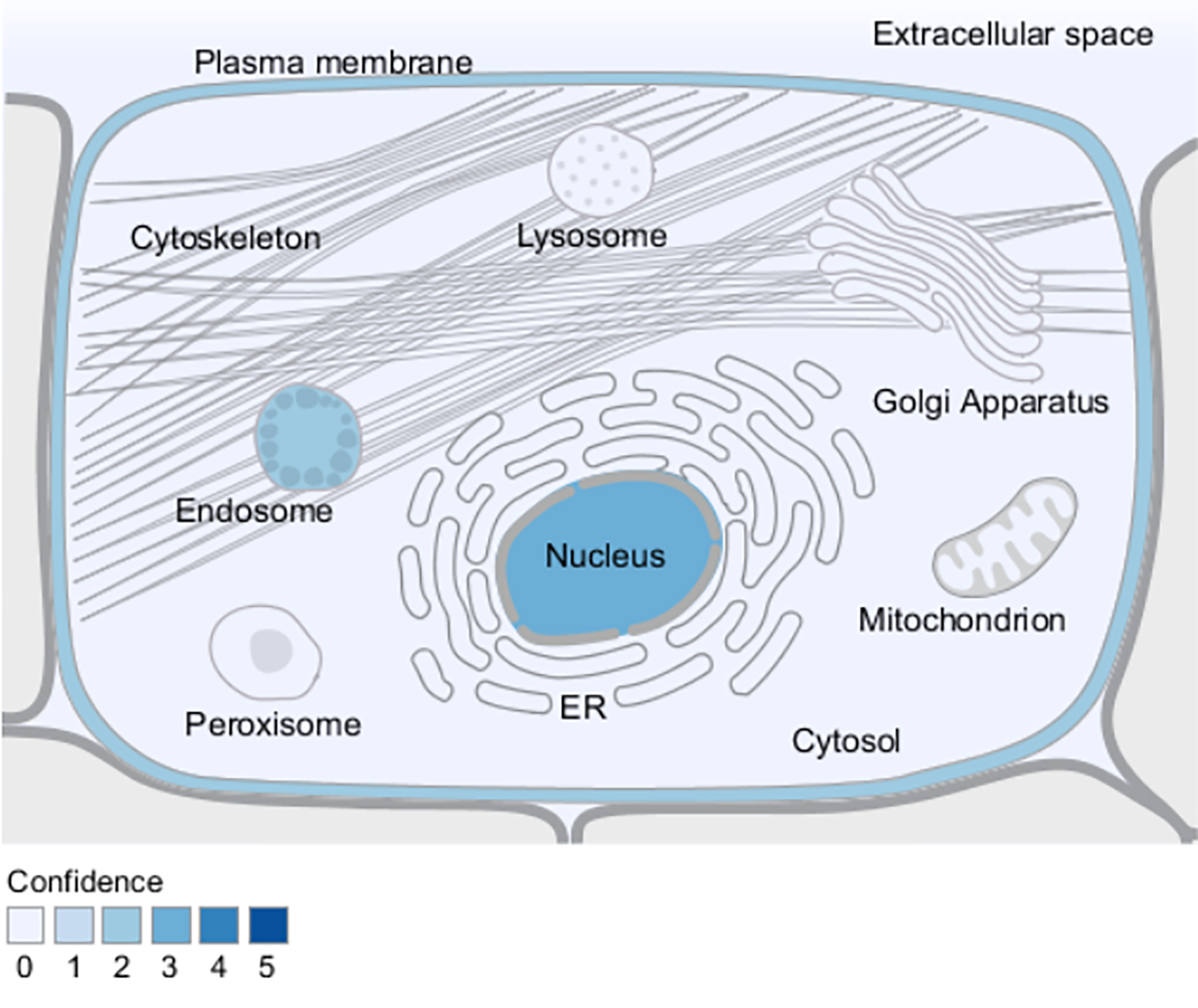

Supplement: Supplementary file 6 [file Image5.TIF]
